# A high-resolution spatial model of the primate gut microbiome defines niche-specific functions and host-adapted microbial metabolism

**DOI:** 10.1080/19490976.2026.2748153

**Published:** 2026-10-16

**Authors:** Shuwen Hou, Yuesong Jiang, Dengwei Zhang, Zhenjiang Xu, Yongxin Li, Xiqiang Liu, Haitao Zhang, Jiachao Zhang, Qixiao Zhai, Chuan Zhang, Xinrui Wang, Shi Huang

**Affiliations:** a Faculty of Dentistry, The University of Hong Kong, Hong Kong SAR, China; b Department of Chemistry, Faculty of Science, The University of Hong Kong, Hong Kong SAR, China; c State Key Lab of Food Science and Technology, Nanchang University, Nanchang, China; d Department of Oral and Maxillofacial Surgery, Nanfang Hospital, Guangzhou, China; e Medical Research Center, Fujian Maternity and Child Health Hospital, College of Clinical Medicine for Obstetrics & Gynecology and Pediatrics, Fujian Medical University, Fuzhou, China; f School of Food Science and Engineering, Key Laboratory of Food Nutrition and Functional Food of Hainan Province, Hainan University, Haikou, China; g Collaborative Innovation Center of One Health, Hainan University, Haikou, China; h State Key Laboratory of Food Science and Resources, Jiangnan University, Wuxi, China; i School of Food Science and Technology, Jiangnan University, Wuxi, China; j Shenzhen Institute of Research and Innovation, The University of Hong Kong, Shenzhen, China

**Keywords:** Non-human primates, gut microbiome, microbiome, bile acids, gastrointestinal tract, *Segatella copri*, isoalloLCA

## Abstract

The spatial organization of the gastrointestinal microbiome is shaped by strong physicochemical gradients along the gut, yet many region-associated microbial niches remain poorly resolved in humans. Here, we used the rhesus macaque (*Macaca mulatta*) as a spatially resolved primate model to profile 148 luminal and mucosal samples across the gastrointestinal tract using shotgun metagenomics, 2bRAD-M sequencing, and targeted bile acid metabolomics, complemented by comparative human datasets. Microbial communities exhibited strong spatial stratification along the gastrointestinal tract, while host species explained greater variation than anatomical location in our comparative datasets. Despite broad phylum-level similarity between humans and monkeys, substantial divergence emerged at species and functional levels. Monkey-associated *Segatella copri* encoded expanded carbohydrate-active enzyme (CAZyme) repertoires specialized for degradation of complex plant polysaccharides, consistent with dietary and other host-associated adaptation. Along the gastrointestinal tract, microbial load and diversity followed pronounced spatial gradients, although the low-biomass stomach retained unexpectedly high taxonomic and functional diversity. Mucosal communities largely mirrored luminal microbiota at broad taxonomic scales but exhibited distinct species-level signatures associated with niche-specific functional specialization. The monkey large intestine was further enriched in the immunomodulatory bile acid isoallo-lithocholic acid (isoalloLCA), with candidate biosynthetic genes identified in previously uncharacterized microbial lineages. Together, these findings establish a high-resolution framework of the primate gut microbiome and reveal how spatial organization and host-associated selection shape microbial metabolism and host–microbe interactions relevant to gut health.

## Introduction

The gastrointestinal (GI) tract hosts a complex and spatially structured microbial ecosystem, where gradients in pH, oxygen, nutrients, and bile acids create distinct niches.^1^ These region-specific communities modulate key physiological functions, including digestion and metabolic signaling.^2^ Precise profiling of microbial distribution along the GI tract is therefore essential for understanding the patterns of host-associated microbiomes.

Despite this, human microbiome studies so far have relied on a limited set of sample sites, primarily saliva and stool, which offer incomplete representations of the oral and gut microbiota.^2–4^ Comprehensive human GI sampling is invasive and ethically challenging.^5^ Capsule-based and post-mortem sampling have each improved access to human intestinal sites but remain limited by uncertain sampling location and pre-mortem interventions.^2^ ^,^ ^5^ Moreover, mucosal microbiomes, tightly associated with host tissues, remain poorly characterized due to technical challenges of high contamination in human DNA.^6^

Non-human primates, particularly rhesus macaques (*Macaca mulatta*), overcome these barriers. Their close phylogenetic and physiological resemblance to humans,^7^ ^,^ ^8^ combined with the feasibility of whole‑tract sampling, enables spatial mapping of gastrointestinal microbial communities and comparative analysis of conserved and host-specific features across primates. Despite their wide use in biomedical research,^9^ studies of the monkey GI microbiome remain limited. Previous work profiled distal gut mucosa and lumen by 16S rRNA gene sequencing.^10^ We extend these efforts to establish a spatially resolved, whole-GI tract microbiome atlas at the species level through shotgun metagenomics and 2bRAD-M.

Here, we hypothesized that the primate gut microbiome comprises spatially distinct communities whose functions are constrained by local physiological conditions. Host-specific factors could drive divergence at the taxonomic and metabolic levels. To test this, we sampled the rhesus macaque gastrointestinal tract at high spatial resolution to capture niche partitioning along its longitudinal and luminal-mucosal axes, using human intestinal communities as a cross-host reference. We systematically examined (i) cross-species conservation versus divergence in community structure, (ii) the spatial organization of luminal and mucosal microbial communities along the GI tract, and (iii) how niche-specific microbial functions are translated into host-relevant metabolic outputs, including bile acid transformation. Together, this work delivers a comprehensive genomic and functional catalog of the primate gut microbiome and establishes a mechanistic framework for dissecting how spatial organization and host selection jointly shape the colonization, metabolic specialization, and host–microbe interactions of gut microbial communities.

## Materials and methods

### Rhesus macaques

A total of four rhesus macaques (*Macaca mulatta*; two 8-y-old and two 20-y-old) were selected for the study (Table S1). Care and sample collection details see Supplementary Methods. All animal procedures were reviewed and approved by the Fujian Maternity and Children's Health Hospital Committee on Animal Ethics (IACUC Issue No.: SFYAEC20241007) and were conducted in accordance with the Regulations on the Administration of Experimental Animals of the People's Republic of China and related institutional guidelines.

### Human participants (public dataset)

For comparative purposes, we downloaded public human GI tract samples. Only three previous studies have successfully performed holistic sampling of human intestines.^2^ ^,^ ^11^ ^,^ ^12^ Among these, we selected the dataset provided by Shalon et al., as Zmora et al. lacked oral samples, and She et al. relied exclusively on 16S rRNA sequencing rather than shotgun metagenomics. Shalon et al. collected luminal content from six body sites (saliva, four intestinal sites of different pH, and stool); raw FASTQ files were downloaded from NCBI BioProject PRJNA822660 and targeted bile acid profiles from Metabolomics Workbench (ST002075). For age- and sex-matched comparison with our four-monkey cohort, we restricted downstream analyzes to four male participants from Shalon et al.: two young adults (ages 20 and 32) and two older adults (ages 63 and 65).

### DNA extraction and shotgun metagenomics sequencing

DNA was extracted from fresh frozen luminal content samples using MagPure Soil DNA KF Kit (Magen Biotech, Guangzhou, China), followed by library preparations using ND617-02 kit (Vazyme, Nanjing, China). Inputs were sequenced on the MGISEQ-T7 platform (BGI, Shenzhen, China), and paired-end reads were generated. 66 metagenomic samples provided an average of 75 million reads. The metagenomic raw data were quality-controlled using Kneaddata (v0.12.0, huttenhower.sph.harvard.edu/kneaddata/). Reads mapped to the rhesus monkey database (Feb. 2019, rheMac10, The Genome Institute at Washington University School of Medicine, NCBI Assembly ID: 2345051) were filtered. The average reads passing filter is 54 million.

## 2bRAD-M sequencing

DNA was extracted from fresh frozen mucosal and blood tissue samples using CretMag Multi Sample DNA Kit (Cret Biotech, Suzhou, China), followed by 2bRAD-M reduced metagenomics sequencing by Qingdao OE Biotech Co., Ltd. (Qingdao, China).^6^ Detailed library preparation protocols and quality control thresholds are provided in Supplementary Methods.

We collected air, scissor, forceps, and monkey skin swabs as negative controls. All negative controls were processed and sequenced alongside biological samples. Taxa present at >5% relative abundance in any of these controls were identified as potential contaminants and were removed from all biological samples prior to any downstream analysis. Relative abundances were subsequently normalized within each sample. Contaminant burden averaged 4.03% per sample but reached 40.13% in one small-intestinal mucosal sample. Sensitivity analysis excluding this sample gave nearly unchanged results for all community-level analyzes.

### Microbial profiling

Taxonomic profiling was performed with Kraken 2 and species-level abundances refined with Bracken.^13^ Eukaryota were removed and bacterial profiles merged into a combined abundance table for downstream analyzes.

MetaPhlAn 4 was used to perform strain-level metagenomic phylogenetic analysis.^14^ HUMAnN 3 was used to profile microbial gene family and pathway abundances.^15^ Human data were re-processed with the same pipeline. Detailed tool versions, and parameters please see Supplementary Methods.

We used 2bRAD-M pipeline (available at https://github.com/shihuang047/2bRAD-M) for the species-level characterization of microbial communities. Detailed bioinformatics rationale please see Supplementary Methods.

### Metagenomic assembly and functional profiling

Metagenomic reads were assembled with MEGAHIT,^16^ aligned to contigs with Bowtie 2,^17^ and binned with MetaBAT 2^18^; bin quality was assessed with CheckM,^19^ yielding 1677 representative MAGs (Fig. S1A, Table S2). MAGs were taxonomically classified with GTDB-Tk^20^ and visualized with ggtree (Fig. S1B).^21^ Genes were predicted with Prodigal^22^ and annotated for CAZymes with dbCAN^23^; CAZyme reference annotations and *S. copri* clade genome resources were obtained from Blanco-Míguez et al. ^24^

A custom DIAMOND database^25^ of isoalloLCA-producing reference enzymes (Sato et al.) was built and queried against MAGs by BLASTp. ARGs were annotated with DeepARG,^26^ and secondary metabolite BGCs were identified with antiSMASH.^27^ Viral contigs were identified with VirSorter2 and VIBRANT,^28^ ^,^ ^29^ merged per sample, and clustered into vOTUs with MMseqs2^30^; vOTU quality was assessed with CheckV.^31^

Homologous protein structures were predicted with ColabFold^32^ and pairwise structural similarity was quantified with US-align.^33^ Full tool versions, database releases, and parameter settings are provided in the Supplementary Methods.

### Cross-platform concordance between 2bRAD-M and shotgun metagenomics

All luminal content samples (*n* = 66) were additionally profiled using 2bRAD-M for direct comparison with shotgun metagenomic profiles. Species-level relative abundance tables generated by the two approaches showed strong concordance across matched samples (Spearman's *ρ* = 0.96, *p* < 0.001, Fig. S1C).

### Microbial load measurement

DNA extracted from 0.025 g luminal content sample with an elution volume of 10 μL was sent to qPCR for microbial load quantification. Detailed qPCR procedure please see Supplementary Methods.

### Statistical analysis

Statistical analysis was conducted with R (v.4.2.2). The profiling results of all monkey and human samples were imported to R in the form of feature tables. Phyloseq package (v.1.42.0) was used for data handling.^34^ Low-abundance (relative abundance < 0.1%) species were filtered using R package metagMisc (v.0.5.0).^35^

Correlation analyzes between microbial absolute abundances and bile acid concentrations were performed using Pearson's correlation in R. Absolute abundance was calculated by multiplying relative abundance by microbial load (copies/g sample) for each taxon. Correlation coefficients and *p*-values were computed for each taxon–metabolite pair. All *p*-values were adjusted for multiple testing using the Benjamini–Hochberg method.

### Diversity and differential abundance analysis

Alpha diversity was calculated using the Shannon index, and beta diversity was estimated using Bray–Curtis dissimilarity, both computed on species-level relative abundance profiles. We performed PERMANOVA and PERMDISP tests based on species-level beta diversity, using the adonis2 and permutest functions in vegan (v.2.6-4).^36^ Host individual was included as a fixed covariate (apart from cross-host PERMANOVA), and marginal effects were tested using 999 permutations restricted within individuals. A principal coordinate analysis (PCoA) plot was generated to visualize the first two principal coordinates with the ggplot2 package in R (v.3.5.2).^37^

Differential abundance analysis of species profiles was carried out using R package MaAsLin3 (v.1.0.2)^38^ to capture both prevalence and abundance shifts. Taxa with prevalence ≥ 20% and abundance ≥ 0.1% were tested with log transformation, and significance was defined as BH corrected *p* < 0.1. Host individual was included as a random effect in all MaAsLin3 models. MetaCyc pathway abundances in each body location were compared using the Kruskal–Wallis test. Differentially abundant pathways were presented on the heatmap using R package pheatmap (v.1.0.12).^39^

### Other downstream analysis

Procrustes analysis was conducted using the procrustes function in R package vegan (v.2.6-10) with Bray–Curtis-based PCoA ordinations as input. The degree of fit was assessed using the *M^2^* statistic, and significance was tested with 999 permutations.

Source tracking analysis was performed using FEAST (v.0.1.0) to estimate the proportion of mucosal microbial communities originating from luminal content.^40^ For each intestinal site, luminal samples were set as sources and mucosal samples as sinks.

### Targeted bile acid profiling

Bile acids were quantified in luminal content and mucosal tissues by targeted UHPLC–MS/MS in MRM mode against internal-standard–normalized calibration curves (Table S3). Extraction protocols, chromatographic gradients, and instrument parameters are provided in Supplementary Methods.

## Results

### Comparative microbiome profiling in humans and monkeys

We collected paired luminal and mucosal samples (*N* = 148) spanning the oral cavity, esophagus, stomach, small intestine, and large intestine of rhesus monkeys (Figure 1A), profiling luminal contents by shotgun metagenomics and mucosal tissues by 2bRAD-M^6^ (Fig. S2A). For cross-species comparison, we re-analyzed capsule-sampled human shotgun data from four age- and sex-matched participants (BioProject PRJNA822660; see Methods).

**Figure 1.**
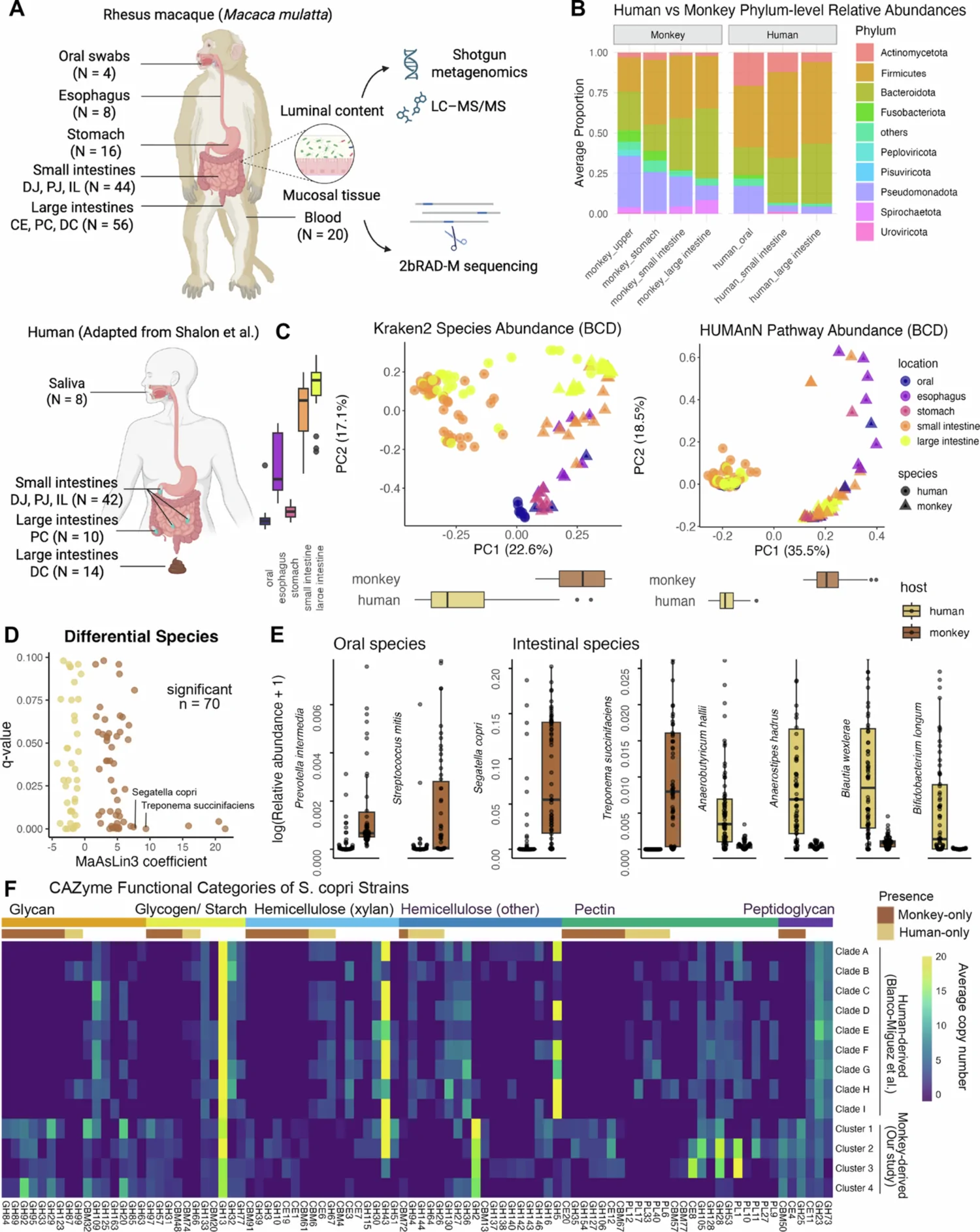
Comparative microbial and functional landscapes of monkey and human intestines. (A) Sample collection sites of four rhesus monkeys and subsequent analysis. Total sample size *N* = 148. For cross-host comparison, publicly available gastrointestinal samples from four age- and sex-matched male human participants from Shalon et al. were analyzed. Abbreviations: Duodenojejunal Flexure (DJ), Proximal Jejunum (PJ), Ileum (IL), Cecum (CE), Proximal Colon (PC), and Descending Colon (DC). Created with BioRender. (https://BioRender.com/z6x1nhn) (B) Stacked bar plot showing the mean relative abundance of microbial phylum across different human and monkey samples. Samples are grouped based on their location. (C) PCoA plot based on Bray–Curtis dissimilarity of species-level microbial composition (left) and pathway abundance (right) in luminal samples of both humans and monkeys. PERMANOVA showed significant effects of host and gastrointestinal location (*p* = 0.001; Table S4). (D) Volcano plot showing robust significant MaAsLin3 results (BH adjusted *p* < 0.1) comparing rhesus macaques and human participants. A total of 70 species met this criterion. (E) Relative abundances of selected differentially abundant species between human and monkey luminal content samples. (F) Heatmap showing the average copy numbers of carbohydrate-active enzyme (CAZyme) families across human-derived *S. copri* clades A-I and monkey-derived clusters 1 to 4. The first upper bar denotes CAZyme functional categories. The second upper bar denotes whether the CAZyme showed in monkey-derived genomes only or human-derived genomes only.

Across GI sites, humans and monkeys showed broadly similar phylum-level compositions (Figure 1B, S2B), both dominated by *Firmicutes* with increasing *Bacteroidota* along the tract. In addition, Shannon diversity in both hosts was highest in the oral cavity, declined in the small intestine, and rebounded in the large intestine (Fig. S2C).

Despite the overall phylum-level similarity, higher-resolution analyzes revealed clear structural differences. Principal coordinate analysis (PCoA) of species-level Bray–Curtis dissimilarities (Figure 1C, Table S4) separated samples primarily by host on PC1 (22.6%) and by GI site on PC2 (17.1%). Hosts thus accounted for more variation than within-tract spatial variation. Human and monkey oral samples clustered closely, suggesting conserved oral microbiomes. Oral, esophageal, and stomach samples clustered together as an upper-GI group, while small and large intestine samples formed distinct clusters.

PERMANOVA (Table S4) confirmed significant effects of host and location (*p* = 0.001), with location explaining less variation in humans (*R* ^2^ = 0.20) than monkeys (*R* ^2^ = 0.42). Pathway abundance profiles showed the same pattern (Figure 1C) that hosts segregate on PC1 (PERMANOVA *p* = 0.001). Along PC2, only those of monkey samples are stratified by GI site, indicating that microbial function varies less across GI locations in humans (*R^2^* = 0.14) than monkeys (*R^2^* = 0.29).

We next identified 70 differentially abundant species between humans and monkeys using MaAsLin3 (Figure 1D, Table S5). Oral pathogens including *Prevotella intermedia*, *Prevotella denticola*, *Prevotella melaninogenica*, *and Streptococcus mitis* were enriched in monkeys. Many species enriched in humans were probiotic, including *Bifidobacterium longum*, *Blautia wexlerae*, *Anaerobutyricum hallii*, and *Anaerostipes hadrus.* These species are linked to gut health through refined-sugar metabolism and short-chain fatty acid production,^41^ ^,^ ^42^ consistent with high-carbohydrate modern diets. In contrast, the monkey gut microbiome was dominated by specialists of plant fiber degradation, most notably *Segatella copri* (formerly *Prevotella copri*) and *Treponema succinifaciens* (Figure 1E).

The above differential abundance findings were robust to participant selection. Repeating the analysis with all 15 Shalon et al. participants recovered the same human–monkey divergence, with effect sizes across species tightly correlated between the two analyzes (Spearman *ρ* = 0.981, Fig. S2D). All key taxa, including *S. copri* and *T. succinifaciens,* remained significant (Fig. S2E).

*S. copri* was the most abundant species in 31 of the 46 monkey small and large intestine samples. Given its prevalence, we further investigated *S. copri*. Monkey-derived *S. copri* strains were assigned to clade K (SGB1626) using MetaPhlAn4 species-specific markers. Screening reconstructed MAGs against the clade K reference recovered 18 *S. copri*–like genomes.

Given that *S. copri* strains vary substantially in their carbohydrate-active enzyme (CAZyme) repertoires,^24^ ^,^ ^43^ we next profiled CAZymes in the monkey-derived MAGs. We annotated CAZymes in our monkey-derived MAGs using dbCAN, followed by Bray–Curtis dissimilarity analysis and k-means clustering, and resolved four distinct CAZyme-defined functional clusters (Fig. S3). For comparison, we retrieved the curated CAZyme profiles of human-associated *S. copri* clades A–I from Blanco-Míguez et al. ^24^. Monkey-derived genomes encoded a substantially broader CAZyme repertoire for degrading complex plant polysaccharides than human-associated clades (Figure 1F).

Several CAZyme families were absent from human-derived *S. copri*, including *α*-glucan targeting enzymes (GH57 amylases, GH31 *α*-glucosidases, CBM48 starch-binding module) and exoglycosidases (GH92 *α*-mannosidases, GH95 *α*-L-fucosidases) that process plant-derived oligosaccharides. Monkey-derived genomes additionally encoded hemicellulases (GH3, GH39, GH10, CE1, CE19, CBM61) for xylan/arabinoxylan breakdown, pectin-degrading enzymes (GH35, CE12), and cell-wall modules (CBM50, CE4). Together, these expansions point to greater plant-polysaccharide degrading potential in non-human primate *S. copri*.

### Spatial stratification of the microbiome along the GI tract

To examine how microbial composition and function are structured along the GI tract, we first quantified microbial load at distinct anatomical sites by 16S rRNA qPCR (Figure 2A). Load was high in the oral cavity, depleted in the esophagus and stomach, rose through the small intestine (notably the ileum), and peaked in the rectum at ~2000-fold the small-intestinal level.

**Figure 2.**
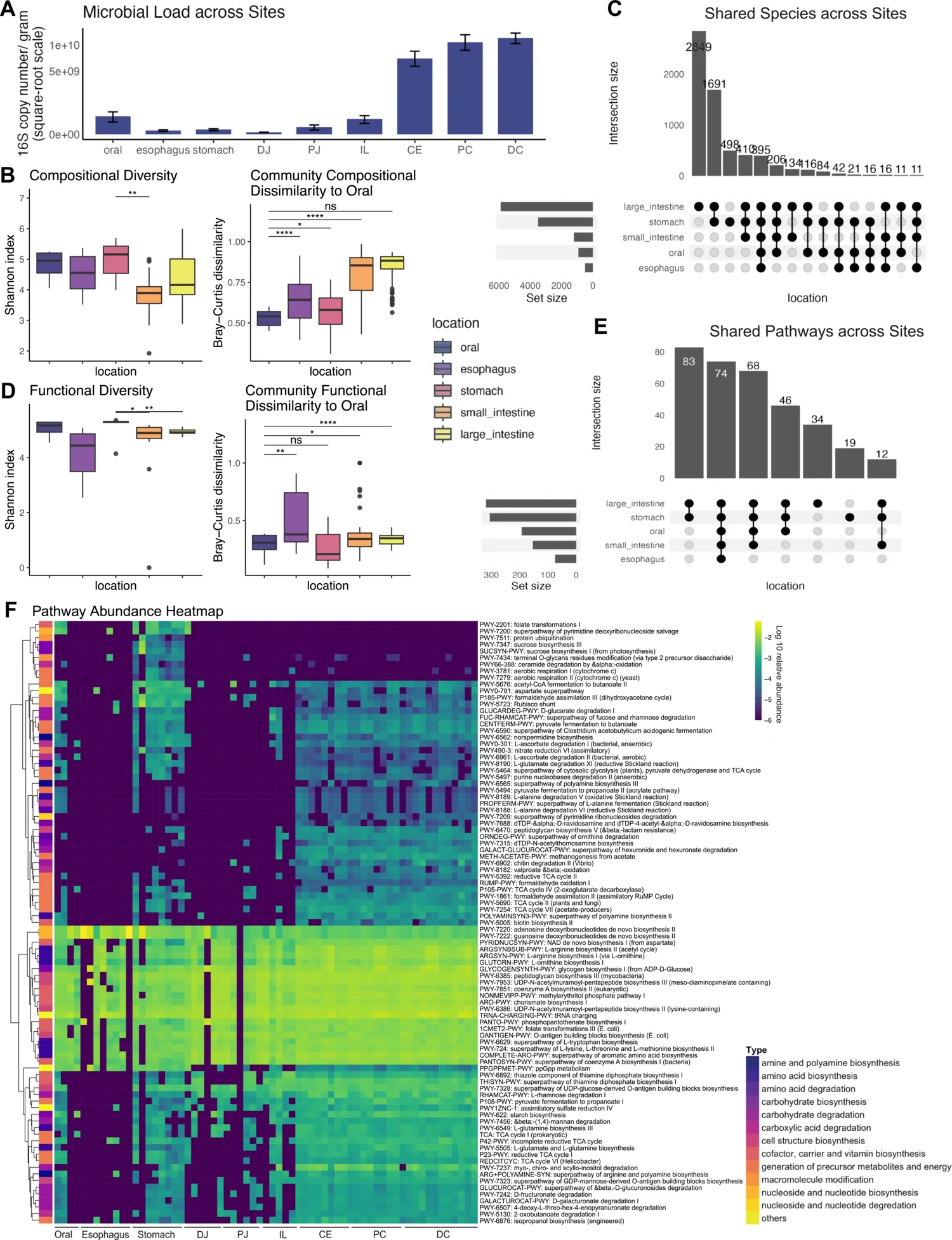
Regional diversity and functional differentiation along the primate GI tract. (A) Bar plots showing microbial load measured by qPCR quantification of 16S rRNA gene copy number per gram sample collected from various regions of the GI tract. Bars show the mean across biological replicates for each region, and error bars represent the standard error of the mean (SEM). (B) Species-level taxonomic diversity across gastrointestinal regions measured by the Shannon index (left) and Bray–Curtis dissimilarity between oral microbial communities and communities at other gastrointestinal regions (right). Significant differences among regions were assessed using Wilcoxon rank-sum test. (C) Upset plot of shared species between different locations (prevalence > 50% in each site). Bars indicate the number of species corresponding to each intersection of gastrointestinal regions. (D) Functional diversity across gastrointestinal regions measured by the Shannon index of MetaCyc pathway abundance (left) and Bray–Curtis dissimilarity between oral and other gastrointestinal regions based on pathway abundance profiles (right). Significant differences among regions were assessed using Wilcoxon rank-sum test. (E) Upset plot of shared pathways between different locations (prevalence > 50% in each site). Bars indicate the number of pathways corresponding to each intersection of gastrointestinal regions. (F) Heatmap (log-scale abundance) of MetaCyc pathways showing groups of pathways with significantly different abundances across oral cavity, esophagus, stomach, small intestine (DJ, PJ, IL), and large intestine (CE, PC, DC) samples (Kruskal‒Wallis test, BH adjusted *p* < 0.01). Pathways are annotated by MetaCyc ontology classification.

Species-level diversity across body sites was assessed by Shannon index (Figure 2B). Diversity was high in the upper GI tract (oral, esophagus, stomach), dropped sharply in the small intestine, and rebounded in the large intestine and stool. This trend paralleled microbial load except in the stomach, which retained high diversity despite low biomass. A Venn diagram revealed substantial overlap in species composition between the stomach and oral cavity. To assess whether this overlap accounted for the observed gastric diversity, we excluded species shared between the oral cavity and stomach and recalculated taxonomic and functional diversity. Following the exclusion, the stomach retained high taxonomic diversity and functional diversity, indicating that the distinct diversity patterns of the gastric microbiome were not solely driven by oral-shared taxa (Fig. S4A).

To further characterize the spatial turnover of microbial communities, we calculated Bray–Curtis dissimilarities between sites. We observed a gradual increase in microbial community dissimilarity along the digestive tract, with esophageal and gastric samples showing the greatest resemblance to oral communities, whereas small and large intestinal samples progressively diverged (Figure 2B).

An upset plot of shared and site-specific taxa (prevalence > 50%; Figure 2C, Table S6) identified a conserved core of 395 species detected across the entire GI tract, being generalists broadly distributed across the GI tract. Representative examples include the metabolically versatile species *Faecalibacterium prausnitzii*, *Bacteroides thetaiotaomicron*, and *Escherichia coli*. A total of 1691 species were shared between the stomach and large intestine, while site-restricted taxa were most diverse in the large intestine, which harbored 2849 unique species—far more than any other site.

Differential abundance analysis across GI sites revealed clear taxonomic stratification, with distinct clusters corresponding to the upper GI tract (oral cavity, esophagus), stomach, and the combined small and large intestines (Fig. S4B). Classic oral pathogens (*Porphyromonas gingivalis, Tannerella forsythia, Fusobacterium nucleatum, Aggregatibacter actinomycetemcomitans, Treponema denticola*) were enriched in the stomach. In contrast, the small and large intestines were dominated by anaerobic fermenters, including *Faecalibacterium prausnitzii*, *Agathobacter rectalis, Anaerobutyricum hallii, Roseburia intestinalis,* and *Segatella copri*, consistent with their roles in polysaccharide fermentation and short‑chain fatty acid production.

We next assessed the functional potential of microbial communities across sites using HUMAnN 3. Interestingly, in contrast to the pronounced taxonomic shifts, functional pathway diversity was relatively uniform across most regions, including the stomach and small intestine (Figure 2D). Bray–Curtis dissimilarity between locations calculated by pathway abundance remained uniformly low across body sites, suggesting that microbial functional profiles are far more conserved than taxonomic profiles.

To further delineate the functional architecture of gastrointestinal microbiomes, we examined pathway overlap across locations using an upset plot (Figure 2E, Table S6). A core set of 74 pathways was present across all sites, forming a conserved functional backbone. Eighty-three additional pathways were shared specifically between the stomach and large intestine, reinforcing the functional parallels between these geographically distant but metabolically similar niches. Site‑specific specialization was also evident. The large intestine exhibited 34 pathways uniquely detected at this site, while the stomach harbored 19 such pathways (Figure 2E), indicating location-associated functional signatures.

To systematically identify region‑specific metabolic functions, we visualized 91 significantly different MetaCyc pathways across locations in a heatmap (Figure 2F, Kruskal–Wallis test, BH adjusted *p* < 0.01). Hierarchical clustering revealed a core biosynthetic module composed mostly of amino acid and cofactor biosynthesis pathways. The remaining differentially abundant pathways showed markedly reduced representation in the esophagus and small intestine. Large intestinal regions were markedly enriched in pathways supporting energy production and metabolic throughput, including TCA cycle (PWY-5690, P105-PWY, PWY-7254), pyruvate fermentation (CENTFERM-PWY, PWY-5494, P108-PWY), and carbohydrate degradation (GLUCARDEG-PWY, GALACT-GLUCUROCAT-PWY, PWY-7456) pathways. Despite low microbial load, the stomach showed unique enrichments for aerobic respiration (PWY-3781, PWY-7279) and terminal O-glycan modification (PWY-7434). Additionally, pathways involved in ceramide degradation (PWY66-388) and folate metabolism (PWY-2201) were also enriched, suggesting microbial adaptation to the stomach’s nutrient-limited and physiologically challenging environment.

Consistent with niche-specific selection pressures, gene-level features encoded in the reconstructed MAGs also exhibited region-specific patterns. Antibiotic resistance gene (ARG) profiles differed markedly between stomach and large intestine, with efflux and *β*-lactam target genes in Neisseria and Moraxella dominating the stomach and intrinsic tolerance and regulatory genes in Clostridia and Lachnospiraceae characterizing the large intestine (Fig. S5A). Biosynthetic gene cluster (BGC) annotations in large-intestine MAGs were enriched for cyclic-lactone autoinducer biosynthesis and signal-transduction domains across phylogenetically diverse taxa, indicating a widespread quorum-sensing trait rather than a lineage-specific one (Fig. S5B). Beyond bacterial genomes, we recovered 257 medium- to high-quality viral operational taxonomic units (vOTUs), including 84 integrated prophages; vOTU profiles showed pronounced inter-individual separation, indicating highly individualized viral reservoirs (Fig. S5C).

### Distinct mucosal and luminal communities

To resolve how microenvironmental gradients shape microbial community composition, we compared luminal and mucosal communities within each anatomical region. PCoA on Bray–Curtis dissimilarity showed samples clustering primarily by anatomical location and secondarily by compartment (mucosal vs. luminal); blood samples separated clearly from all GI sites (Figure 3A). PERMANOVA confirmed that both factors significantly explained community variation (*p* = 0.001), with location accounting for a larger proportion of variance (*R^2^* = 0.252) than compartment (*R^2^* = 0.197). Pairwise PERMANOVA confirmed that anatomical location explained a substantial proportion of community variation (Figure 3A).

**Figure 3.**
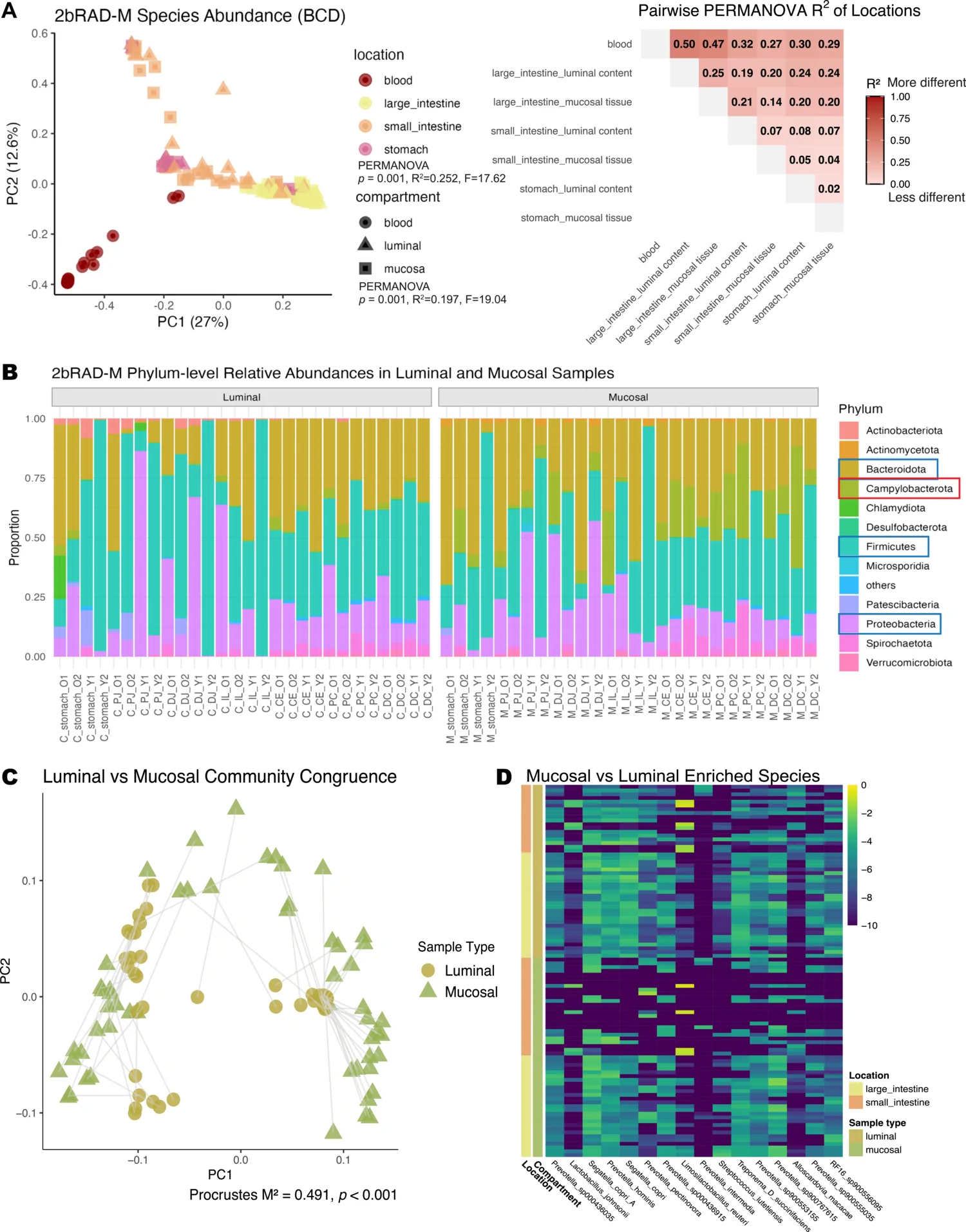
Mucosal–luminal microbial relationships in the monkey gut. (A) PCoA plot based on Bray‒Curtis dissimilarity between microbial composition determined by 2bRAD-M of all monkey samples (PERMANOVA and PERMDISP, *p* = 0.001). The matrix shows pairwise PERMANOVA comparisons of microbial communities across sites and parts based on Bray–Curtis dissimilarity of species-level abundance profiles. Effect size (*R^2^*) represents the proportion of total community variation explained by the grouping factor location. A lower *R^2^* indicates that samples from different locations are more similar relative to variation within locations. (B) Stacked bar plot showing the relative abundance of microbial phylum across different mucosal and luminal content samples. Samples are grouped based on sub-location. Abbreviations: Luminal content (C), Mucosal tissue (M). (C) Procrustes analysis comparing paired mucosal and luminal content microbial communities (Procrustes *M^2^* = 0.491, *p* < 0.001). (D) Heatmap (log-scale abundance) showing species with significantly different abundances between mucosal and luminal samples identified using MaAsLin3 (BH adjusted *p* < 0.1).

Along the GI tract mucosal tissues, large intestine samples formed relatively tight and overlapping clusters, whereas stomach and small intestine samples were more broadly distributed (PERMDISP: LI vs. SI mucosal, F = 83.67, *p* = 0.001; LI vs. stomach mucosal, F = 41.57, *p* = 0.001). SI and stomach mucosal showed comparable dispersion (PERMDISP: F = 0.89, *p* = 0.187). This suggests more heterogeneous mucosal communities in the stomach and small intestine, likely reflecting steep physicochemical gradients that characterize stomach and small intestine, in contrast to the more uniform anaerobic conditions of the large intestine.

To enable direct cross-compartment comparison, all luminal samples (*n* = 66) were also profiled by 2bRAD-M (see Methods). Phylum-level profiles were broadly concordant between matched mucosal and luminal samples (Figure 3B), with Firmicutes, Bacteroidota, and Proteobacteria dominant in both compartments, indicating shared core niches across the luminal–mucosal axis. Compartment-specific signatures were nonetheless evident, most notably Campylobacterota enrichment in the large-intestine mucosa, consistent with its microaerophilic, hydrogenotrophic lifestyle.^44^

To determine whether phylum‑level similarities extended to higher taxonomic resolution, we performed Procrustes analysis on species‑level community profiles. This revealed a significant concordance between the mucosal and luminal communities (Figure 3C, *M^2^* = 0.491, *p* < 0.001), suggesting that while mucosal and luminal compartments harbor distinct microbial profiles, their community structures remain partially aligned. Source tracking analysis (Fig. S6) further revealed that, on average, over 50% of the mucosal microbiota could be traced back to luminal content, suggesting substantial microbial influx from the lumen to the mucosal surface.

Despite the overarching structural congruence, mucosal and luminal niches selected distinct taxa. Differential abundance analysis identified 16 taxa significantly enriched in luminal content, including *Segatella copri* and *Prevotella hominis* (Figure 3D). In contrast, no species reached significance in mucosal samples after multiple-testing correction. Together, these findings are consistent with mucosal niches acting as selective filters that refine luminal community composition through local environmental conditions.

### Distinct regional bile acids patterns and isoalloLCA enrichment

Finally, we asked whether this spatial and host-specific organization affects host metabolism. We profiled bile acids along the monkey GI tract and compared them with human intestinal profiles from Shalon et al.^2^ (Figure 4A). Total bile acid concentrations rose along the monkey GI tract from the small to the large intestine, opposite the progressive decline reported along the human GI tract (per-subject log slopes; Welch's t-test, t = 7.02, *p* = 0.001).

**Figure 4.**
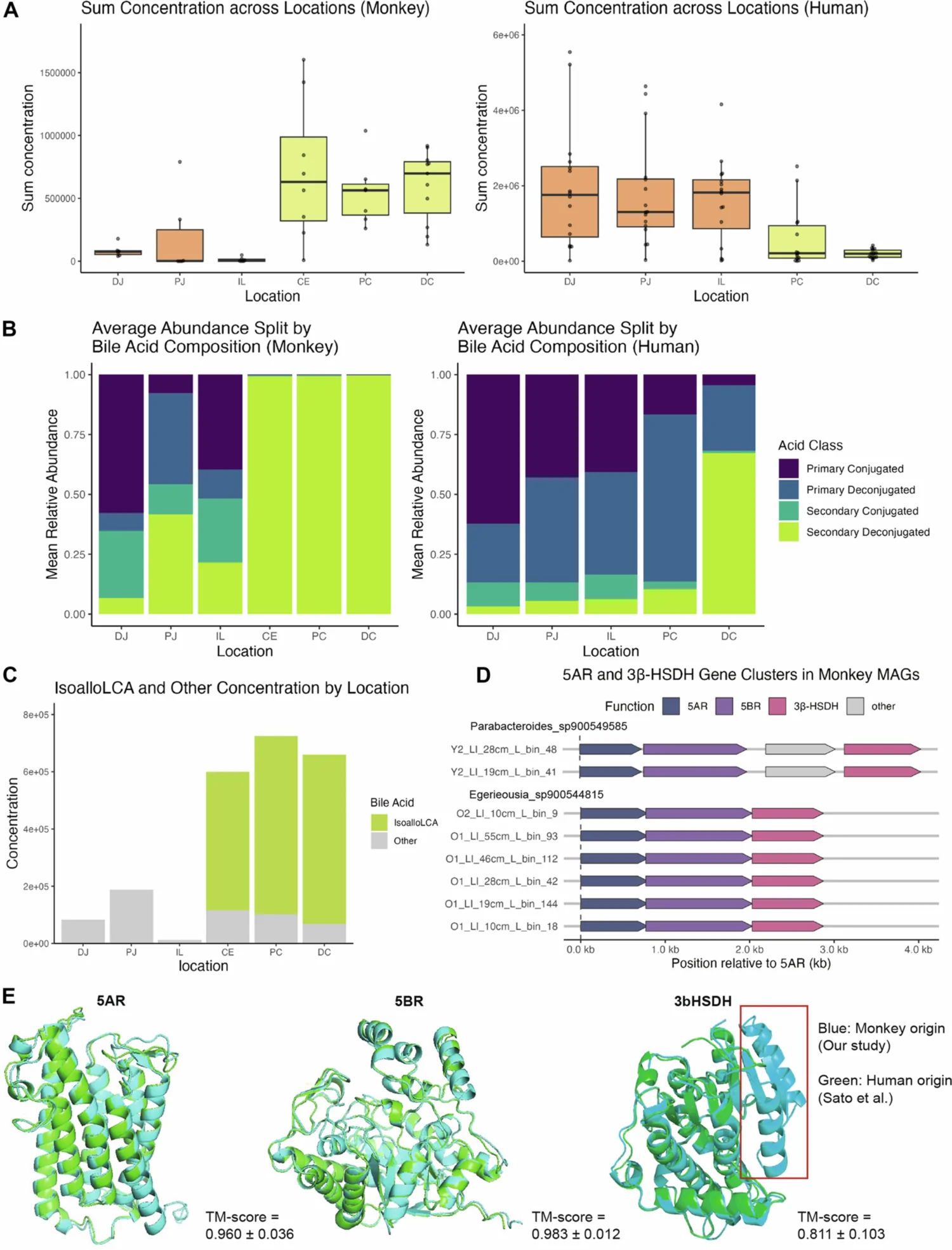
Spatial distribution of bile acids and microbial associations. (A) Box plots showing total bile acid concentrations (ng per gram sample) across intestines in monkeys (left, sample *n* = 46) and humans (right, sample *n* = 81). Per-subject log10 slopes across regions were compared between hosts by two-sided Welch's t-test (*p* = 0.001). (B) Stacked bar plot showing relative bile acid composition by chemical class across GI tract in monkeys (left) and humans (right). (C) IsoalloLCA concentrations across monkey GI compartments. (D) Organization of 5AR, 5BR, and 3β-HSDH in *Egerieousia* sp900544815 and *Parabacteroides* sp900549585 MAGs. Arrows indicate ORFs scaled to genomic coordinates and oriented by transcriptional direction, centered on 5AR. (E) Predicted protein structures of 5AR, 5BR, and 3β-HSDH encoded in monkey-associated (blue) and human-associated (green) genomes. Models were generated using AlphaFold2 and are shown in ribbon representation. The mean and standard deviation of pairwise TM-score between human- and monkey-derived structures were annotated.

The relative composition of bile acids also differed between hosts. In monkeys, secondary deconjugated species dominated the large intestine, while the small intestine carried a mixed profile of primary and secondary conjugated forms (Figure 4B). Humans, by contrast, showed only a gradual decline in primary conjugated and rise in secondary deconjugated species toward stool.

The most abundant bile acid in the monkey large intestine was isoallo-lithocholic acid (isoalloLCA), accounting for a median of 89% of total measured bile acids across all monkey large intestine samples (Figure 4C), whereas it was undetectable in the Shalon et al. human dataset. IsoalloLCA was previously reported as enriched in the feces of healthy human centenarians^45^ and exhibits antimicrobial activity against gram-positive bacteria and promotes regulatory T cell differentiation.^46^

To identify candidate isoalloLCA producers in the monkey gut, we screened our MAGs for the three enzymes required for isoalloLCA biosynthesis^45^ (Fig. S7). MAG hits were assigned by GTDB-tk to *Parabacteroides* sp900549585 or *Egerieousia* sp900544815 (order Bacteroidales), indicating that isoalloLCA biosynthesis in the monkey gut is likely carried out by lineages distinct from the previously reported human producers (Figure 4D, Table S7).

We next asked whether enzyme structural variation contributes to the elevated isoalloLCA concentrations observed in monkeys. Using AlphaFold2, we predicted protein structures of homologous enzymes from human- and monkey-origin microbes and compared their structural similarity by pairwise TM-score (Figure 4E, Fig. S8). Enzyme sequences from human-origin microbes were downloaded from Sato et al.^45^ 5AR and 5BR proteins showed high structural similarity between monkey and human homologs (average TM-scores 0.96 and 0.98, respectively). In contrast, 3β-HSDH was less conserved, with an average TM-score of 0.81. Structural inspection identified two additional *α*-helices in the monkey homolog, a feature that could enhance thermostability and catalytic efficiency. Gene-expression differences could also contribute to the observed enrichment and remain to be tested experimentally.

## Discussion

In this study, we demonstrated that the primate gut microbiome is structured by spatially distinct niches that impose conserved functional constraints, while host-specific factors drive divergence in microbial composition and metabolic functions. By combining whole-tract luminal and mucosal sampling in rhesus monkeys with comparative human data, we provide a spatially resolved framework to examine how microbial communities are organized along the GI tract. Monkey-associated *S. copri* carried an expanded CAZyme repertoire, reflecting host-associated functional divergence shaped by diet together with host evolutionary history. Along the GI tract, microbial load and diversity followed pronounced spatial gradients, yet the low-biomass stomach retained unexpectedly high taxonomic and functional diversity. Monkey large intestine was markedly enriched in the immunomodulatory bile acid isoalloLCA, with candidate biosynthetic genes identified in previously uncharacterized lineages.

Cross-species comparison revealed that capsule-derived human intestinal samples exhibit weaker spatial structuring than anatomically defined monkey samples, consistent with the inherent uncertainty in spatial localization of capsule sampling. This underscores the value of the rhesus macaque as a spatially resolved model for primate GI microbiome profiling.

Host-specific factors exert strong selection pressures on the gut microbiome, driving adaptations in microbial genetic and metabolic capacities.^47^ ^,^ ^48^ The monkey gut was dominated by fiber‑associated taxa such as *Segatella copri* and *Treponema succinifaciens*, which are rare or absent in modern Westernized human populations but persist in traditional societies. *S. copri* is reported to be strongly shaped by habitual diet (plant-rich versus Westernized diets), with distinct CAZyme repertoires spanning arabinan, xylan, xyloglucan, levan, and inulin utilization.^49^ Our cross-host comparison further reveals that monkey-derived *S. copri* genomes encode expanded sets of amylases, hemicellulases, pectinases, and exoglycosidases, exceeding the CAZyme repertoires of human-associated *S. copri* reference genomes. This broader enzymatic repertoire likely reflects long-term adaptation to the fiber-rich diets of non-human primates within their distinct evolutionary, physiological, and environmental context. A parallel pattern holds for *T. succinifaciens*, a spirochete that degrades hemicellulose and pectin and ferments the released sugars to succinate and acetate. Found to be enriched in monkeys in our dataset, *T. succinifaciens* was reported to be abundant in traditional human societies, where populations consume fiber-rich diets.^50^ These monkey-associated lineages thus offer valuable genomic resources for investigating the mechanisms of complex carbohydrate and fiber metabolism.

We next examined how microbial communities are spatially distributed along the monkey GI tract. Despite the general assumption of low diversity in the stomach,^12^ we observed low total microbial biomass yet unexpectedly high compositional and functional diversity in stomach microbial communities. Moreover, the persistence of high diversity after excluding oral-shared taxa indicates that transient microbes from oral input alone does not account for this pattern. Beyond species diversity, differential pathway abundance analyzes identified unique functions of the stomach in aerobic respiration, ceramide degradation, and folate metabolism. These findings challenge the view of the stomach as a microbiologically simple, function-limited niche, instead portraying it as a metabolically specialized environment shaped by microbial transmission and physiological selection.

Small intestine, including both luminal contents and mucosal tissue, differed markedly from other GI sites. It is now widely accepted that small intestine is not a scaled-down colon. Instead, it is the primary site of digestion and absorption which features faster transit and a thinner mucus barrier, creating a distinct microbial ecosystem with closer host contact.^51^ Prior monkey microbiome analysis showed that although stool composition closely mirrors the colonic lumen and mucosa, it is substantially less representative of small intestine communities, particularly mucosal taxa. Consistent with this, the small intestine harbored the lowest microbial load and species diversity along the monkey GI tract, and presented a distinct community pattern that mirrored neither the stomach nor the large intestine. Instead, it was broadly dispersed in composition and lacked the site-specific functional signatures that defined both flanking regions.

Another central finding is the decoupling of taxonomic composition from metabolic function across spatial niches. Despite pronounced species turnover along the GI tract, a core set of metabolic pathways was maintained across sites, suggesting functional redundancy. In highly recombining gut microbial communities, adaptive metabolic genes may spread across diverse taxa via horizontal transfer or homologous recombination, allowing functionally important pathways to be maintained despite species turnover.^52^ In our study, this redundancy was especially evident in the stomach and large intestine, where phylogenetically diverse microbial taxa encoded overlapping metabolic functions, likely facilitated by horizontal gene transfer. Such redundancy likely enhances microbiome resilience, maintaining functional stability under perturbations and contributing to microbiome robustness despite taxonomic turnover.^53^ Spatial niches thus constrain metabolic requirements while permitting taxonomic flexibility, linking spatial organization to community stability.

The niche-specific pattern of the GI tract microbiome extends beyond central metabolism to accessory genomic features. Both ARG and BGC profiles exhibited clear region-specific patterns, with distinct resistance gene repertoires partitioned between the stomach and large intestine and quorum-sensing biosynthetic domains convergently enriched across diverse colonic lineages. In contrast, viral community composition was structured primarily by host individual rather than GI location, likely contributing to inter-host variability in microbial function and host–microbe interactions.

The mucosal interface represents a critical site of host‑microbe interaction, yet its characterization has been hampered by host DNA contamination. Our mucosal profiling using 2bRAD-M method demonstrated similar community structures with luminal counterparts in terms of phylum-level composition, yet species-level analyzes revealed clear differentiation. This is consistent with previous studies showing that luminal or fecal communities can broadly approximate mucosal microbiome composition, while direct mucosal sampling captures finer-scale taxonomic and functional variation.^54^ Such differentiation likely reflects the distinct ecological conditions across the lumen–epithelium axis, including gradients in nutrient availability, oxygen exposure, mucus-derived substrates, and host immune activity.^55^ The intestinal lumen predominantly favors taxa specialized in polysaccharide fermentation and short-chain fatty acid production, as reflected by the enrichment of *S. copri* and *Prevotella hominis*. In contrast, no mucosa-enriched species was observed. This asymmetric pattern involves distributed taxonomic shifts or the selective reduction of species favored in the lumen, rather than enrichment of a small, consistent set of mucosa-specific species. Thus, although luminal profiles approximate mucosal composition broadly, species-level mucosal sampling remains essential to capture niche-specific signatures.

Beyond their spatial organization, gut microbes influence host physiology by transforming host-derived molecules such as bile acids. In the monkey large intestine, we observed a sharp increase in isoalloLCA concentration, a metabolite previously associated with healthy human aging and regulatory T‑cell induction.^45^ Its non-detection in our human comparison dataset suggests either host‑specific production or differential microbial selection. We further identified candidate producer genomes (*Parabacteroides* and *Egerieousia* sp.) harboring co‑localized biosynthetic genes, and comparative structural modeling indicated that monkey‑associated 3β‑HSDH variants are potentially more thermostable than their human counterparts. This provides a functional link between microbial community and host metabolic outputs, demonstrating how spatially and host-specific microbiome organization translates into physiologically relevant metabolites. The pattern of host-specific taxa and metabolic outputs we observe is consistent with the long-term host–microbe co-diversification documented across primates, and extends that framework by showing that host-driven divergence is unevenly distributed across GI niches rather than uniform along the tract.

A major strength is the anatomically precise, whole-tract sampling strategy that is often impractical in humans. Our study also leveraged high-resolution mucosal profiling using the 2bRAD-M method, effective in characterizing low-biomass microbial communities, to achieve high spatial resolution of the primate mucosal gut microbiome. Furthermore, all resources generated in this study have been made publicly available, including all reconstructed MAGs, predicted protein catalogs, CAZyme/PUL, viral sequences, ARG, and BGC annotations, as well as targeted bile acid measurements. These datasets, deposited in Zenodo, enable future researchers to directly use and extend the genomic insights of the non-human primate spatial microbiome.

A notable limitation is the small sample size due to the scarcity of dissected rhesus monkey specimens. Accordingly, the human-monkey differences reported here should be interpreted as cohort-level observations, and further validation is needed. Another limitation is the potential batch effect between the public human dataset and our own. Though we minimized it by processing all samples through an identical bioinformatic pipeline, differences between sample processing and sequencing batches may still exist. While rhesus monkeys offer valuable physiological similarities to humans, direct extrapolation of findings must be approached with caution due to inherent interspecies differences in immune function, diet, and gut physiology. Future studies could involve culturing monkey-derived isolates and conducting intervention experiments to determine their causal contributions to polysaccharide utilization and bile acid metabolism.

## Conclusions

By integrating spatially resolved microbiome and metabolome data across luminal and mucosal compartments, this study establishes a high-resolution compositional and functional framework for primate gut microbial communities. The framework advances our understanding of how spatial heterogeneity along the GI tract shapes the composition, metabolism, and host–microbe interactions. Furthermore, our findings identify specific microbial lineages that warrant further functional study. The expanded CAZyme repertoire of *S. copri* in monkeys identifies a fiber-associated lineage relevant to host adaptation, while the candidate isoalloLCA biosynthetic genes recovered from *Parabacteroides* and *Egerieousia* sp. nominate putative producer genomes for this immunomodulatory metabolite. Together, these represent starting points for mechanistic research of gut fiber and bile acid metabolism and future intervention studies for human gut health.

## Supplementary Material

Supplementary Methods.docx Supplementary Methods.docx

Supplemental Figures.pdf Supplemental Figures.pdf

Supplementary Information.docx Supplementary Information.docx

Supplementary Material Tables.xlsx

## Data Availability

Our sequence data are available on the National Genomics Data Center (NGDC) under BioProject PRJCA052625 (https://ngdc.cncb.ac.cn/bioproject/browse/PRJCA052625). Codes used to generate all plots are available at https://github.com/HuangShiLab/Monkey_spatial_microbiome Genome and functional annotation resources are available at https://zenodo.org/records/17597478.
