# Supplementary material for: A high-resolution spatial model of the primate gut microbiome defines niche-specific functions and host-adapted microbial metabolism
Source: Gut Microbes. 2026 Oct 16;18(1):2748153. doi: 10.1080/19490976.2026.2748153 (PMC13682396; doi:10.1080/19490976.2026.2748153)
Supplement: Supplementary file 1 [file KGMI_A_2748153_SM0571.pdf]

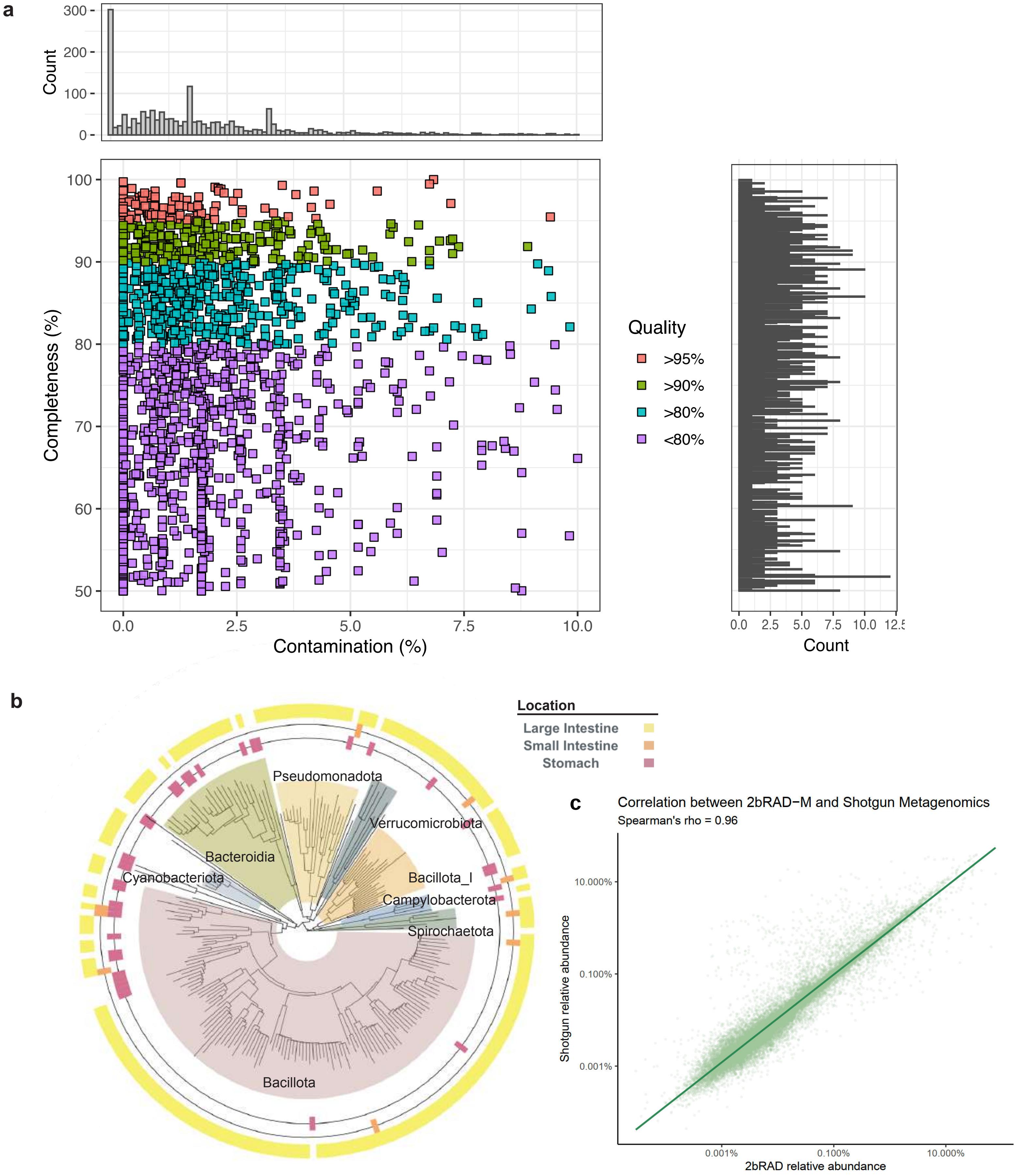


**Figure S1. Quality assessment and phylogenetic overview of reconstructed monkey gut MAGs.**
(A) Quality evaluation of 1,677 metagenome-assembled genomes (MAGs) using CheckM. Colors indicate completeness categories, and marginal histograms show the distribution of completeness and contamination levels. (B) Phylogenetic tree of genera detected across the monkey digestive tract based on shotgun metagenomic sequencing. The outer ring annotates sampling locations, and phyla are color-coded with taxonomic labels. (C) Species-level relative abundances obtained from 2bRAD-M and shotgun metagenomic sequencing were compared across paired luminal content samples (N = 66). Each point represents a species-level abundance measurement from the same sample profiled by both methods. Abundances were log-transformed for visualization.

a

| Figure | Sample Type | Sequencing Method | Profiling Pipeline | Data Level | Used For |
|---|---|---|---|---|---|
| Fig 1C | Luminal | Shotgun metagenomics | Kraken2 + Bracken | Phylum abundance | Relative abundance |
| Fig 1C | Luminal | Shotgun metagenomics | Kraken2 + Bracken | Species abundance | PCoA (Bray–Curtis) |
| Fig 1C | Luminal | Shotgun metagenomics | HUMAnN3 | Pathway abundance | PCoA (Bray–Curtis) |
| Fig 2B | Luminal | Shotgun metagenomics | Kraken2 + Bracken | Species abundance | Shannon diversity |
| Fig 2D | Luminal | Shotgun metagenomics | HUMAnN3 | Pathway abundance | Shannon diversity |
| Fig 3A | Mucosal | 2bRAD-M | 2bRAD-M pipeline | Species abundance | PCoA (Bray–Curtis) |
| Fig 3B | Luminal + Mucosal | 2bRAD-M | 2bRAD-M pipeline | Species abundance | Relative abundance |

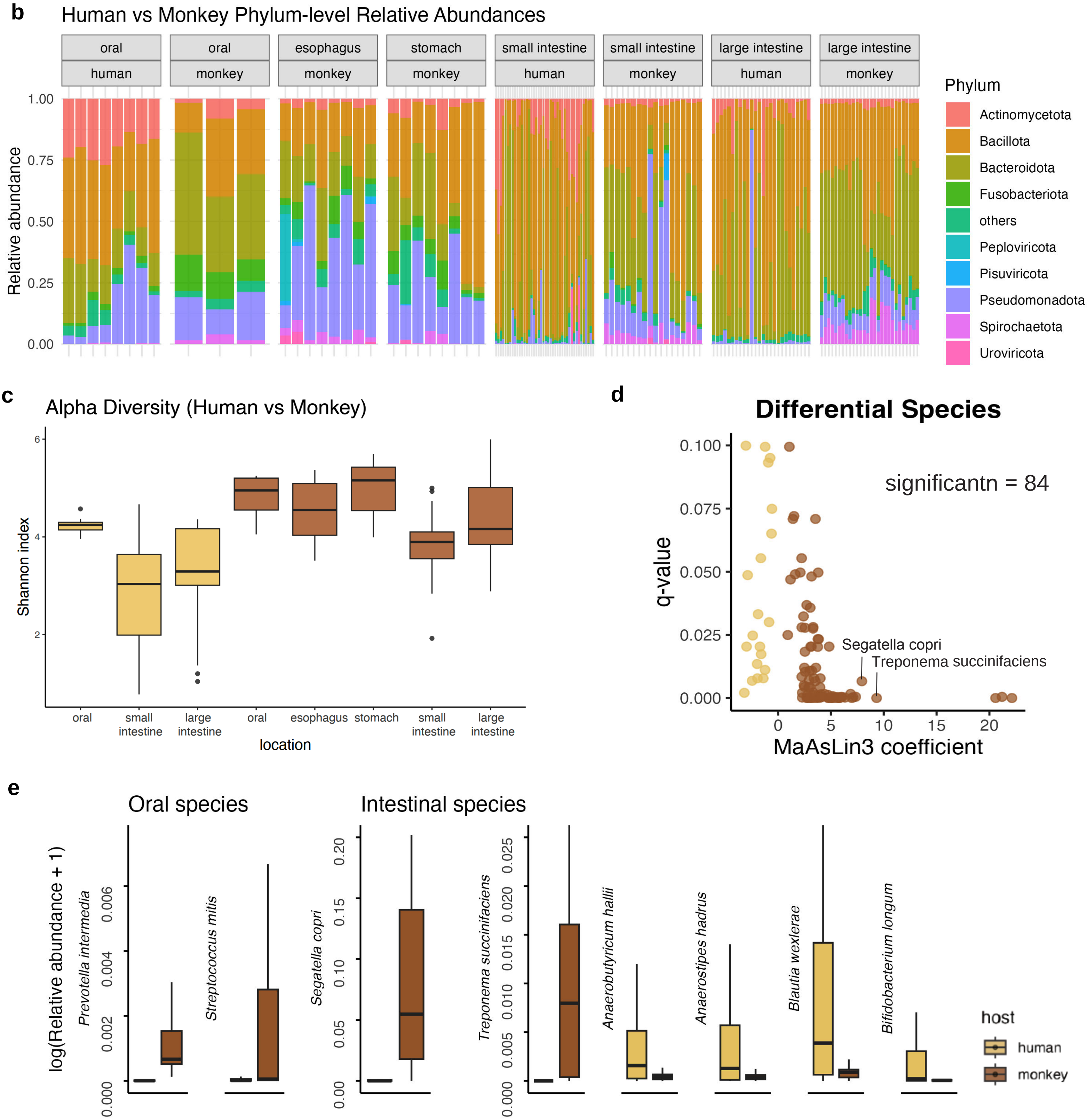


**Figure S2. Cross-species comparison of microbial composition and differentially abundant taxa.** (A) Summary table of sequencing methods together with their sample type and analytical pipelines used for each figure panel (B) Stacked bar plots showing relative abundances of microbial phyla across human and monkey samples, with each column representing one sample. (C) Shannon diversity indices across gastrointestinal sites in humans and monkeys. (D) Volcano plot displaying differentially abundant species identified by Maaslin3 comparing rhesus macaques with 15 human participants from the Shalon et al. cohort (n = 620 samples). (E) Keystone species exhibiting significant differential abundance between human and monkey luminal samples.

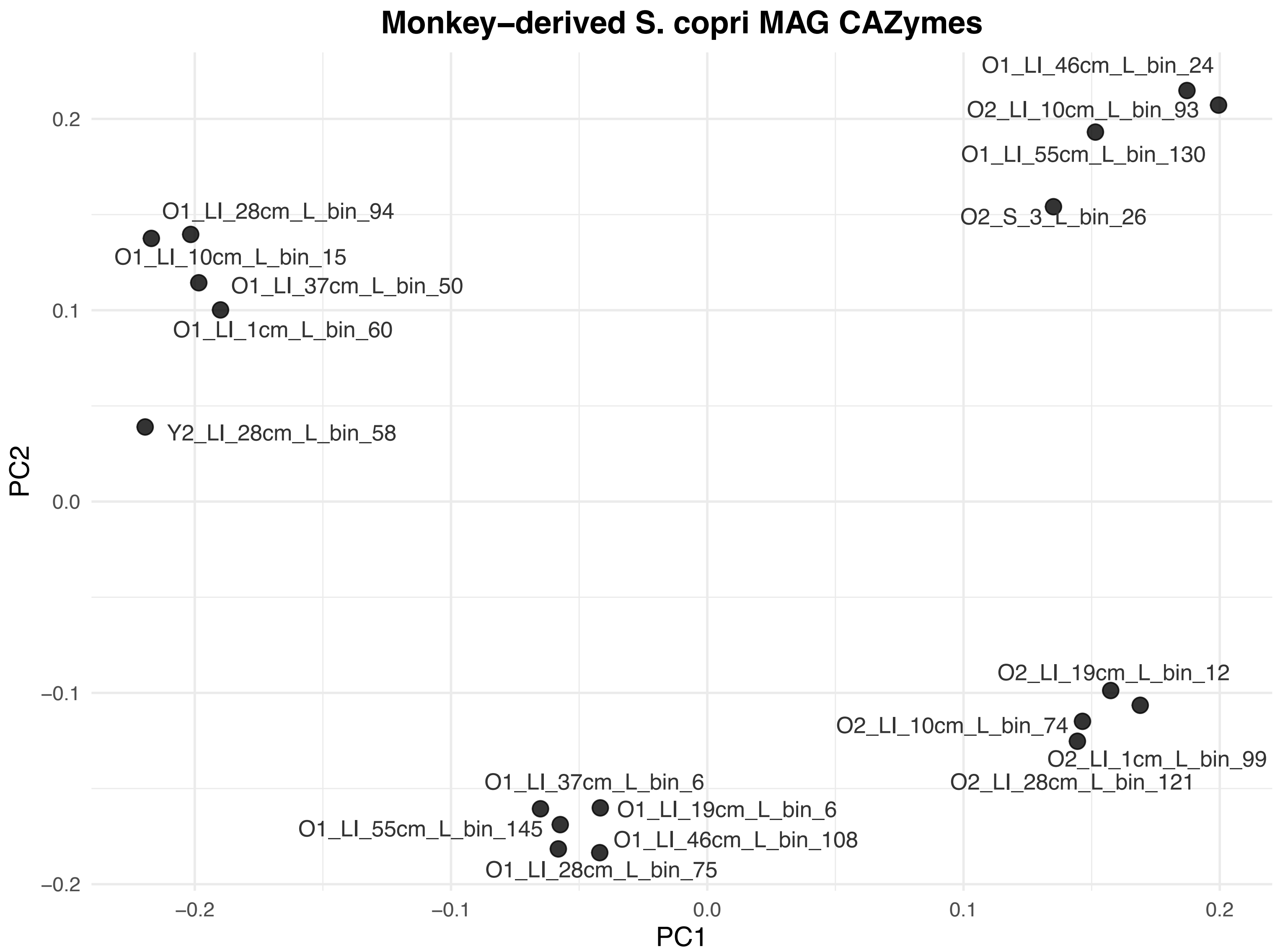


**Figure S3. CAZyme repertoire divergence among Segatella copri genomes**.
Principal coordinates analysis (PCoA) based on Bray–Curtis dissimilarity of CAZyme family abundances across 18 S. copri MAGs recovered from rhesus monkeys. Each point represents one MAG (labeled by bin ID). Separation along PC1 and PC2 reflects divergence in carbohydrate-active enzyme composition among genomes.

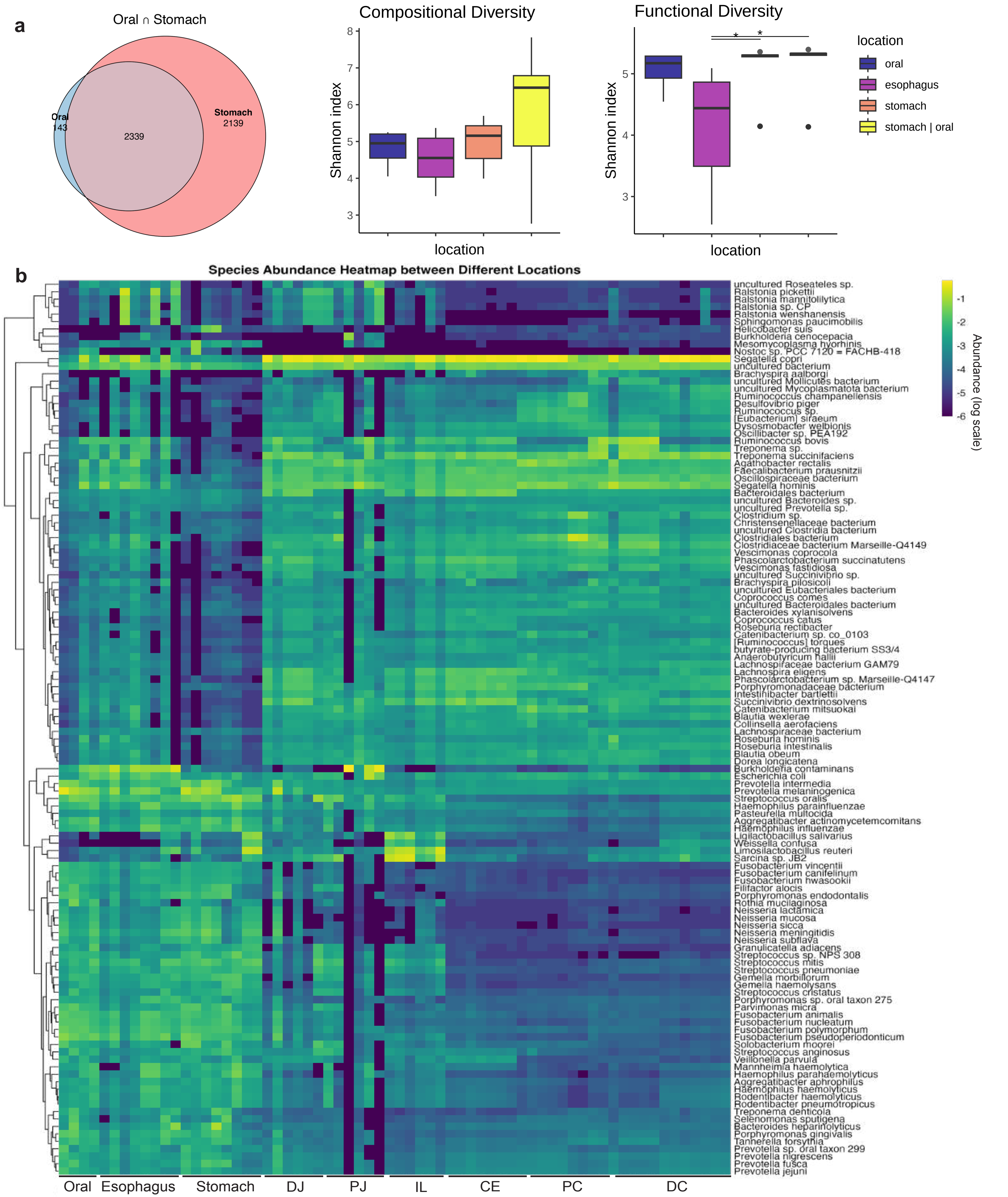


**Figure S4. Spatial distribution and site-specific microbial signatures along the monkey gastrointestinal tract**

(A) Venn diagrams illustrating shared microbial species between oral cavity and stomach. A prevalence threshold of 0.5 was applied. Species-level taxonomic diversity and pathway-level functional diversity across regions. “Stomach | oral” represents gastric profiles after excluding overlapping oral and stomach species.(B) Heatmap of species withsignificantly different abundances across oral cavity, esophagus, stomach, small intestine (DJ, PJ, IL), and large intestine (CE, PC, DC) samples (Kruskal–Wallis test,BH adjusted $p < 0.01$).

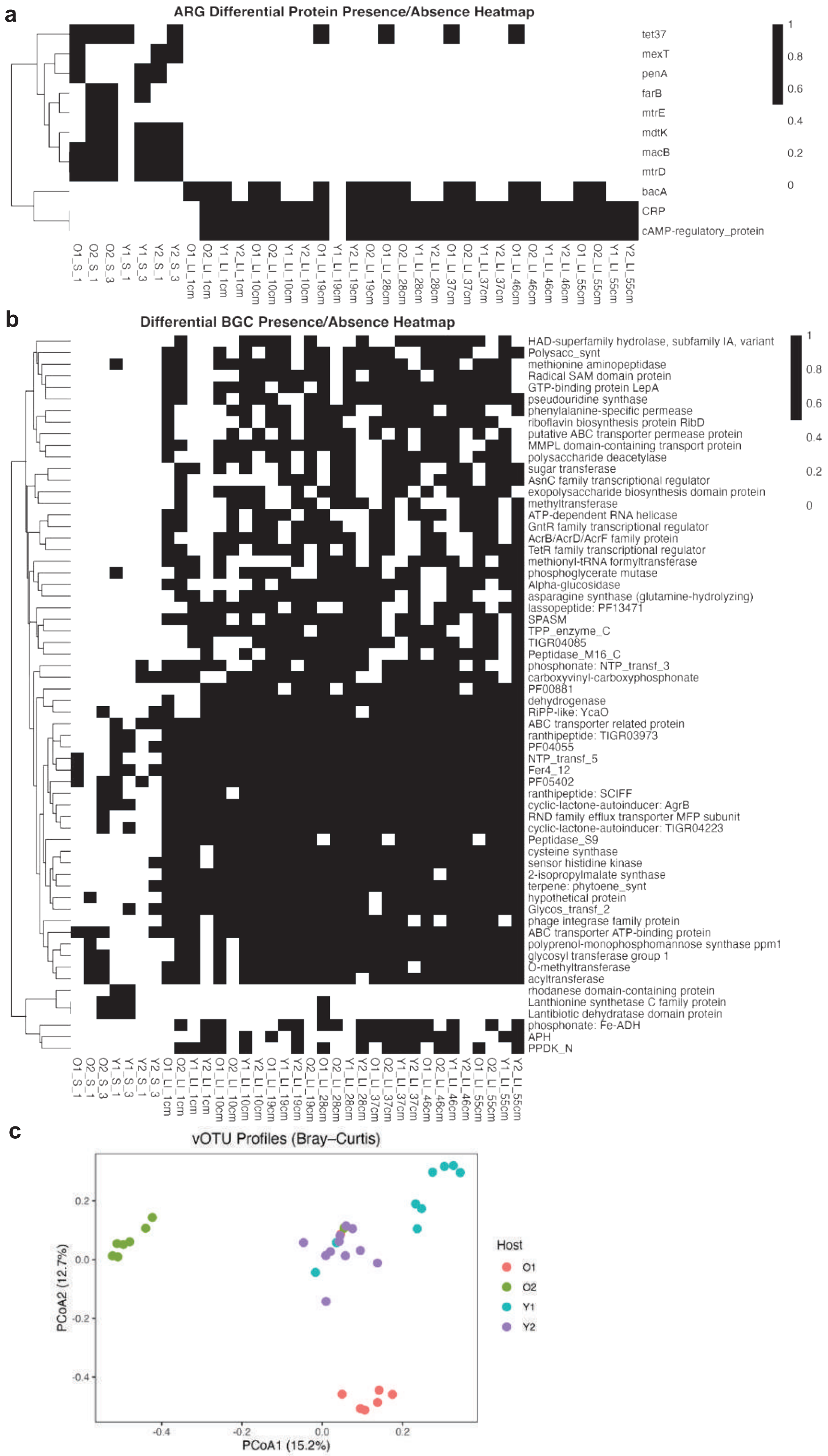


**Figure S5. Differential distribution of antibiotic resistance genes, biosynthetic gene clusters, and viral communities.** (A) Presence–absence heatmap of significantly different antibiotic resistance gene (ARG) protein products between stomach and large intestine samples (chi-square test, BH adjusted $p < 0.05$). (B) Presence–absence heatmap of significantly different biosynthetic gene cluster (BGC) products between stomach and large intestine samples (chi-square test, BH adjusted $p < 0.05$). (C) PCoA of viral operational taxonomic unit (vOTU) community composition based on Bray–Curtis dissimilarities calculated from presence–absence profiles. Each point represents a sample and is colored by host.

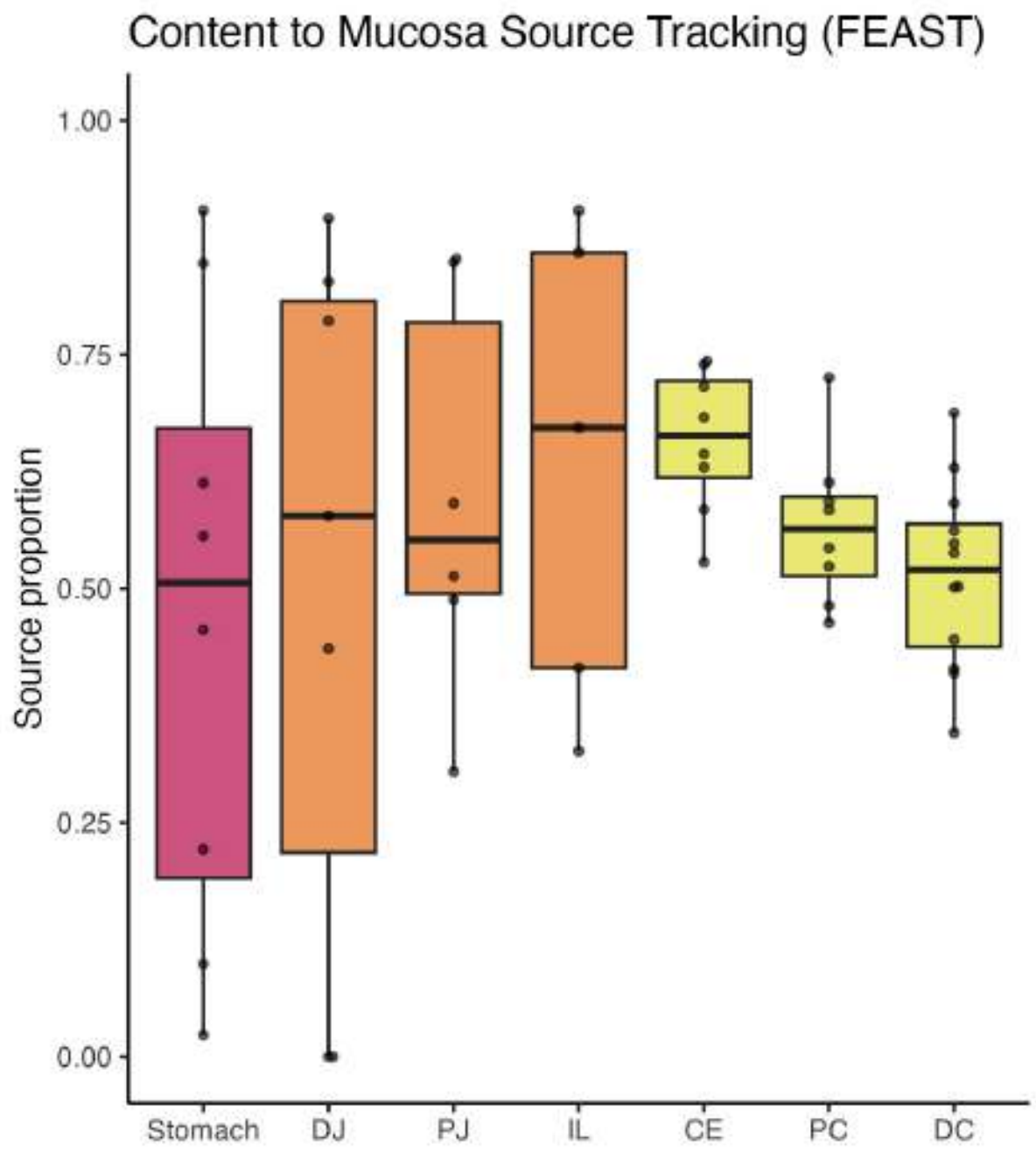


**Figure S6. Luminal-to-mucosal microbial source tracking across intestinal regions.**
Source tracking analysis estimating the proportion of mucosal microbial communities derived from corresponding luminal contents across different intestinal locations.

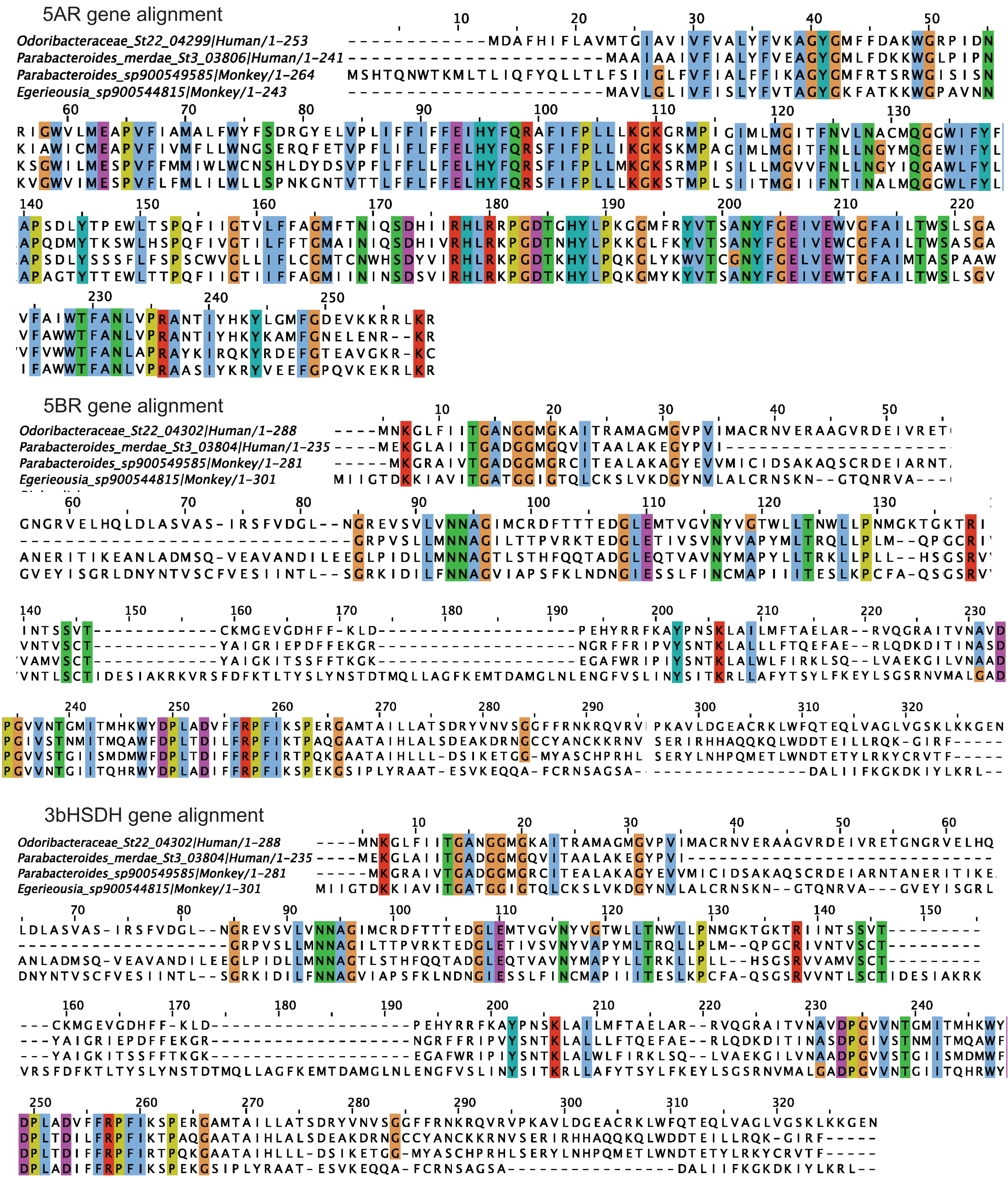


**Figure S7. Comparative sequence alignment of isoalloLCA biosynthetic enzymes from human and monkey microbiomes.** Multiple sequence alignments of key enzymes involved in isoallo-lithocholic acid (isoalloLCA) biosynthesis, including 5α-reductase (5AR), 5β-reductase (5BR), and 3β-hydroxysteroid dehydrogenase (3β-HSDH), comparing homologs from human- and monkey-derived microbial genomes. Human genomes were obtained from the Sato et al. dataset. Amino acids are color-coded by physicochemical properties, and conserved residues are highlighted.

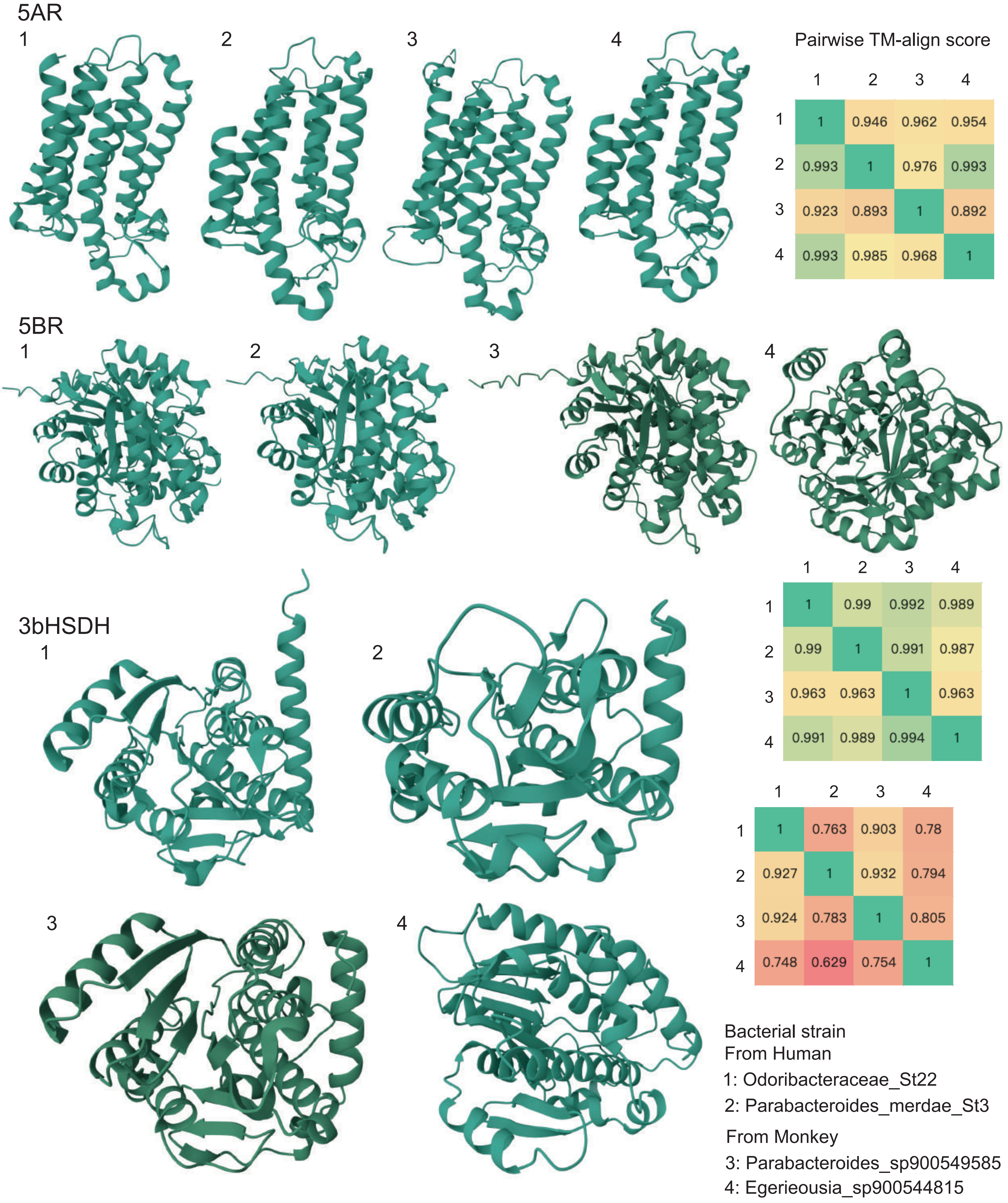


**Figure S8. Predicted structural conservation of isoalloLCA biosynthetic enzymes across host species.**
AlphaFold2-predicted tertiary structures of 5AR, 5BR, and 3β-HSDH from human-origin strains (Odoribacteraceae St22 and Parabacteroides merdae St3) and monkey-origin strains (Parabacteroides sp900549585 and Egerieousia sp900544815), illustrating structural conservation of isoalloLCA biosynthetic enzymes across host species.
