# Supplementary material for: A high-resolution spatial model of the primate gut microbiome defines niche-specific functions and host-adapted microbial metabolism
Source: Gut Microbes. 2026 Oct 16;18(1):2748153. doi: 10.1080/19490976.2026.2748153 (PMC13682396; doi:10.1080/19490976.2026.2748153)
Supplement: Supplementary file 2 [file KGMI_A_2748153_SM0570.docx]

**Supplementary Methods**

**Rhesus macaques**

Rhesus macaques were housed and cared for according to ethical guidelines for the treatment of non-human primates, particularly the rhesus macaque (*Macaca mulatta*), with appropriate veterinary care provided at Fujian Maternity and Children's Health Hospital, Fujian, China. Animals were maintained under controlled environmental conditions and managed in accordance with institutional animal care and welfare standards. Room temperature was maintained at 18–26 °C and relative humidity at 40–70%, under a 12 h light/dark cycle (lights on 08:00–20:00), with adequate ventilation and scheduled cleaning and disinfection. Cages were furnished with perches, swings, hanging rings, toy balls and mirrors to support resting and play behaviour. Animals were pair- or group-housed, had ad libitum access to clean drinking water, and were fed a nutritionally balanced, species-appropriate standard commercial diet three times daily, supplemented once daily with fresh, high-quality fruit and vegetables. Health and welfare checks were performed daily by a veterinarian and trained animal care staff, covering general health status, behaviour, and food and water intake; any abnormality prompted immediate veterinary intervention.

No antibiotics were administered before sacrifice. For euthanasia, each rhesus macaque was anesthetized by intramuscular injection of ketamine hydrochloride (8 mg/kg). After a deep surgical plane of anesthesia was confirmed by loss of the palpebral and pedal withdrawal reflexes, the animal was euthanized by exsanguination through the inguinal (femoral) artery. All anesthesia and euthanasia procedures were performed by trained personnel under veterinary supervision and in accordance with applicable veterinary guidelines, including the American Veterinary Medical Association (AVMA) Guidelines for the Euthanasia of Animals. Post-sacrifice, a series of mucosal tissue and luminal content samples were collected. Swabs were taken from the tongue. Tissue and content samples were taken from two sites (4 cm apart) in the esophagus, two sites in the stomach, five sites in the small intestine, and seven sites in the large intestine, with sampling points spaced evenly along the organ's length. Luminal content was collected first, followed by mucosal tissue sampling after washing with PBS. All samples were stored at −80°C immediately after collection to preserve their integrity for downstream analyses.

**Taxonomic and functional profiling**

Taxonomic profiling was performed with Kraken 2 (v.2.1.2) against the "core_nt" database (released September 2024) using default parameters (--confidence 0, k-mer length 35, minimizer length 31, spaces 7). Read-level classifications were re-estimated to species-level relative abundances with Bracken (v.2.9) using a read length of 150 bp and a minimum read threshold of 10 (-r 150 -l S -t 10). Features assigned to Eukaryota were removed, and the resulting bacterial profiles from all samples were merged into a single abundance table for downstream analyses. In parallel, strain-level metagenomic phylogenetic profiling was performed with MetaPhlAn 4 (v.4.1.0; ChocoPhlAn database vJun23_CHOCOPhlAnSGB_202403, released 20 February 2024) using default parameters with --add_viruses disabled and --unclassified_estimation enabled. Microbial gene-family and pathway abundances were profiled with HUMAnN 3 (v.3.8) against the ChocoPhlAn nucleotide database (v201901_v31) and the UniRef90 translated search database (v201901b), using DIAMOND (v.2.0.15) for translated alignment with default identity and coverage thresholds (--translated-identity-threshold 80, --translated-query-coverage-threshold 90, --translated-subject-coverage-threshold 50). Gene-family abundances were normalized to copies per million (CPM) with humann_renorm_table, and pathway abundances were regrouped to MetaCyc reactions. Human shotgun metagenomes were re-processed from raw reads with the identical pipeline and parameters to ensure cross-host comparability.

**2bRAD-M Sequencing**

Genomic DNA obtained was subjected to digestion with 4 U of Type IIB restriction enzyme (BcgI) for a duration of three hours at 37°C. A reaction mixture was prepared containing 10 μL of the digested product, T4 DNA Ligase Buffer, 1 mM ATP (NEB), 0.2 μM of adaptors (Ada1 and Ada2), and 800 U of T4 DNA ligase (NEB) for a ligation reaction that lasted 16 hours, followed by enzyme inactivation through heating at 65°C for 20 minutes. For DNA sequencing, PCR amplification was performed in a 40 μL reaction volume. The PCR mix included 7 μL of ligated DNA, 1× Phusion HF buffer, 0.1 μM of Illumina primers, 0.3 mM dNTP, and 0.4 U of Phusion high-fidelity DNA polymerase (NEB). PCR was carried out using a DNA Engine Tetrad 2 thermal cycler (Bio-Rad) for 16 to 28 cycles with the following settings: initial denaturation at 98°C for 5 seconds, annealing at 60°C for 20 seconds, extension at 72°C for 10 seconds, and a final extension at 72°C for 10 minutes. Target bands around 100 bp were cut from an 8 % (wt/vol) polyacrylamide gel, and the DNA was diffused in nuclease-free water for 12 hours at 4°C. Sample-specific barcodes were added through PCR using primers with platform-specific barcodes. The 40 µL PCR mixture contained 50 ng of PCR product from the gel, 0.2 μM of each primer, 0.6 mM dNTP, Phusion HF buffer, and 0.8 U of Phusion high-fidelity DNA polymerase (NEB). The PCR products were purified with the QIAquick PCR Purification Kit (Qiagen, Valencia, CA), followed by sequencing using the Illumina NovaSeq 6000 platform.

Clean reads were obtained by filtering out reads containing more than 8% ambiguous bases, removing low-quality reads with a quality score lower than Q30, and performing paired-end read merging. This generates an average of 7 million reads.

**MAG assembly, annotation, and downstream analysis**

Metagenomic samples with > 3 million reads after filtering were assembled with MEGAHIT (v.1.2.9). Reads were aligned to assembled contigs with Bowtie 2 (v.2.5.0), and contigs were binned with MetaBAT 2 (v.2.12.1), producing 4,815 genome bins. Bin quality was assessed with CheckM (v.1.2.2); bins with > 50% completeness and < 10% contamination were retained, yielding 1,677 representative MAGs. Taxonomic classification was performed with GTDB-Tk (v.2.4.0) using the R220 database release (24 April 2024) via the classify_wf command, and the resulting phylogeny was visualized with ggtree (v.3.16.0). Protein-coding genes were predicted with Prodigal (v.2.6.3). CAZymes were annotated with dbCAN (v.4.1.4, database V14, 2025-08-26). Curated CAZyme reference annotation profiles and genome resources for human-associated S. copri clades A– (> 50% completeness and < 5% contamination) were obtained from Blanco-Míguez et al. Reference sequences of isoalloLCA-producing enzymes were taken from the supplementary materials of Sato et al., and a custom protein database was built with DIAMOND (v.2.1.9) using the makedb command. Homologous enzymes were identified with DIAMOND BLASTp (--evalue 1e-12 --id 30 --query-cover 60). ARGs were annotated with DeepARG (v.1.0.2), filtering hits with < 80% identity or < 90% bitscore. Secondary metabolite BGCs were identified with antiSMASH (v.7.0.1), retaining only hits in the 'biosynthetic' category. Viral contigs were predicted with VirSorter2 (v.2.2.4) and VIBRANT (v.1.2.1), merged per sample, and clustered into vOTUs at 95% nucleotide identity with ≥ 85% alignment coverage using MMseqs2 (v.18.8cc5c); vOTU representatives were quality-assessed with CheckV (v.1.0.3) in end-to-end mode. Predicted homologous protein sequences were submitted to ColabFold v1.5.5 (AlphaFold2 with MMseqs2 BATCH) with default parameters, and pairwise structural similarity was quantified with US-align (v.20241108).

**2bRAD-M Profiling**

This bioinformatics workflow relies on the pre-established 2bRAD tag database (2bTagDB), which harbors species-specific molecular markers derived from an extensive microbial genome dataset retrieved from GTDB v214. Sequencing reads were electronically digested to fragments containing BcgI recognizing sites. Microbial species present in each microbiota sample were identified via the Genome Taxonomy Database (GTDB), and their relative abundances were quantified by normalizing the sequencing coverage of single-copy, species-specific genetic markers. Specifically, the mean read coverage of 2bRAD markers corresponding to each species was computed to represent the relative number of microbial individuals of that species in the sample. Subsequent calculation of relative abundance was performed by dividing this species-specific coverage value by the total read coverage of all identified microbial species in the respective sample.

**Microbial load measurement**

qPCR amplification was performed on a Light-Cycler Roche 480 instrument (Roche Molecular Systems, Switzerland). The reaction system had a total volume of 10μL, including 5μL 2×TB Green II (Takara, Beijing, China), 0.2μL forward and reverse primers (10μM), and 1μL template DNA. The absolute abundances of bacteria were determined using the 16S rRNA gene V4-V5, using the primer pair 515F (GTGCCAGCMGCCGCGG) and 907R (CCGTCAATTCMTTTRAGTTT). The thermal cycling conditions were as follows: an initial denaturation step at 95°C for 30 s, followed by 40 cycles of 95°C for 5 s, 55°C for 30 s, 72°C for 30 s. All standard curves were prepared by a 10-fold gradient dilution method for plasmid DNA, and the R2 values of the standard curves of bacteria were greater than 0.997. After qPCR, amplification and melting curves were examined to ensure quality, with a single melting peak confirming specificity. Cp values were obtained using LC480 software. The coefficient of variation (CV) was calculated for each set of triplicate reactions; data with CV < 5 % were considered valid. A standard curve was generated by plotting the log10 of known copy numbers against average Cp values, yielding a linear equation with slope between -3.9 and -3.3 and *R²* ≥ 0.99. Absolute copy numbers in samples were determined by substituting Cp values into this equation and reverse-transforming the result from log10. The average copy number of the triplicates was reported as the final absolute abundance (copies/g sample) for each sample.

**Metabolite extraction and sample preparation**

Biological samples were accurately weighed and transferred into pre-cooled 1.5 mL Eppendorf (EP) tubes (−20 °C). Two small steel beads and 600 μL of methanol containing 1 mM butylated hydroxytoluene (BHT) and internal standards: cholic acid-2,2,4,4-d4, glycocholic acid-(glycine-1-¹³C), and L-2-chlorophenylalanine, were added to each tube. The mixture was homogenized in a grinder (45 Hz, 2 min), then subjected to ultrasonic extraction in an ice bath for 10 min. Subsequently, the mixture was centrifuged at 13,000 rpm for 10 min at 4 °C. The supernatant was collected and transferred to brown vials, which were stored at −80 °C until instrumental analysis.

All extraction reagents were pre-cooled at −20 °C prior to use. A pooled quality control sample was prepared by combining equal volumes of extracts from all biological samples, with each QC sample having the same volume as individual samples (**Table S7**).

**Targeted bile acid quantification by UPLC–MS/MS**

Bile acid profiling was performed using a Nexera UHPLC LC-30A system (Shimadzu) coupled to a Phenomenex Kinetex C18 column (2.1 mm × 100 mm, 2.6 µm). The injection volume was 5 µL, the flow rate was 0.45 mL/min, and the column temperature was maintained at 45 °C. The mobile phases consisted of water with 0.1% formic acid (A) and methanol–acetonitrile–isopropanol (1:1:1, v/v/v) containing 0.1% formic acid (B). The gradient program was as follows: 0–0.5 min, 80% A; 0.5–1.5 min, 80%–62% A; 1.5–12 min, 62%–50% A; 12–17.5 min, 50%–5% A; 17.5–19 min, 5% A; and 19.01–20 min, re-equilibration at 80% A.

Mass spectrometric detection was conducted on an AB SCIEX SelexION™ Triple Quad™ 5500 system equipped with an electrospray ionization (ESI) source operated in both positive and negative ion modes. The parameters were as follows: curtain gas 35 psi, collision-activated dissociation (CAD) set to medium, ion spray voltage −4500 V (negative mode) or +5500 V (positive mode), source temperature 450 °C, Gas1 55 psi, and Gas2 55 psi.

**Bile Acids Quantification and Data Processing**

Targeted bile acids were analyzed in multiple reaction monitoring (MRM) mode. In this mode, precursor ions were selected, fragmented by collision-induced dissociation, and characteristic product ions were monitored to ensure specificity and reproducibility. Peak integration was performed using SCIEX OS-MQ software under default settings, with manual inspection where necessary. Calibration curves were generated by serial dilution of mixed standards, and quantification was based on the ratio of analyte to internal standard peak areas. Concentrations were calculated using the regression equations derived from the calibration curves, corrected for dilution factors, and normalized to the original sample weight or volume. Final metabolite concentrations were expressed as ng/g for solid samples, ng/mL for liquid samples, or ng per 10^6 cells for cellular samples.
