# Supplementary material for: A high-resolution spatial model of the primate gut microbiome defines niche-specific functions and host-adapted microbial metabolism
Source: Gut Microbes. 2026 Oct 16;18(1):2748153. doi: 10.1080/19490976.2026.2748153 (PMC13682396; doi:10.1080/19490976.2026.2748153)
Supplement: Supplementary file 3 [file KGMI_A_2748153_SM8586.docx]

**SUPPLEMENTAL INFORMATION**

**Table S1. Sample metadata for rhesus macaque and human gastrointestinal samples.**

**Table S2. Summary statistics of metagenome-assembled genomes (MAGs).**

**Table S3. Quality-control summary for targeted bile acid LC–MS/MS measurements.**

**Table S4. PERMANOVA of community composition by host and gastrointestinal location.**

**Table S5. Differentially abundant species between humans and rhesus macaques.**

**Table S6. UpSet plot membership lists for shared and unique features across sites.**

**Table S7. Candidate genes involved in isoallo-lithocholic acid biosynthesis.**
